# Genome-wide association analysis in dogs implicates 99 loci as risk variants for anterior cruciate ligament rupture

**DOI:** 10.1371/journal.pone.0173810

**Published:** 2017-04-05

**Authors:** Lauren A. Baker, Brian Kirkpatrick, Guilherme J. M. Rosa, Daniel Gianola, Bruno Valente, Julia P. Sumner, Wendy Baltzer, Zhengling Hao, Emily E. Binversie, Nicola Volstad, Alexander Piazza, Susannah J. Sample, Peter Muir

**Affiliations:** 1 Comparative Orthopaedic Research Laboratory, School of Veterinary Medicine, University of Wisconsin-Madison, Madison, Wisconsin, United States of America; 2 Department of Animal Sciences, College of Agricultural and Life Sciences, University of Wisconsin-Madison, Madison, Wisconsin, United States of America; 3 Department of Dairy Sciences, College of Agricultural and Life Sciences, University of Wisconsin-Madison, Madison, Wisconsin, United States of America; 4 Department of Veterinary Clinical Sciences, School of Veterinary Medicine, Louisiana State University, Baton Rouge, Louisiana, United States of America; 5 Department of Clinical Sciences, College of Veterinary Medicine, Oregon State University, Corvalis, Oregon, United States of America; Universita degli Studi di Roma Tor Vergata, ITALY

## Abstract

Anterior cruciate ligament (ACL) rupture is a common condition that can be devastating and life changing, particularly in young adults. A non-contact mechanism is typical. Second ACL ruptures through rupture of the contralateral ACL or rupture of a graft repair is also common. Risk of rupture is increased in females. ACL rupture is also common in dogs. Disease prevalence exceeds 5% in several dog breeds, ~100 fold higher than human beings. We provide insight into the genetic etiology of ACL rupture by genome-wide association study (GWAS) in a high-risk breed using 98 case and 139 control Labrador Retrievers. We identified 129 single nucleotide polymorphisms (SNPs) within 99 risk loci. Associated loci (*P*<5E-04) explained approximately half of phenotypic variance in the ACL rupture trait. Two of these loci were located in uncharacterized or non-coding regions of the genome. A chromosome 24 locus containing nine genes with diverse functions met genome-wide significance (*P* = 3.63E-0.6). GWAS pathways were enriched for c-type lectins, a gene set that includes aggrecan, a gene set encoding antimicrobial proteins, and a gene set encoding membrane transport proteins with a variety of physiological functions. Genotypic risk estimated for each dog based on the risk contributed by each GWAS locus showed clear separation of ACL rupture cases and controls. Power analysis of the GWAS data set estimated that ~172 loci explain the genetic contribution to ACL rupture in the Labrador Retriever. Heritability was estimated at 0.48. We conclude ACL rupture is a moderately heritable highly polygenic complex trait. Our results implicate c-type lectin pathways in ACL homeostasis.

## Introduction

Anterior cruciate ligament (**ACL**) rupture can be devastating and life changing, particularly in young adults, as there is a 78% risk of knee arthritis after ACL rupture that is not influenced by surgical treatment [1]. The incidence of ACL rupture is high; it is estimated at 33.3–36.9/100,000 person years [2,3]. About 80,000 ACL graft surgeries are performed per year in the USA [4], with annual costs of approximately $1 billion [4]. A non-contact mechanism explains most ACL ruptures, with rupture typically occurring during landing or pivoting movements [3]. Risk of contralateral rupture is also high at 11.8% of patients [5]. Physiological and anatomic factors, such as intercondylar notch shape and posterior tibial slope influence disease risk and risk of ACL rupture. Subsequent contralateral ACL rupture is more frequent in females [6]. Although the underlying mechanism has not been fully defined, it is widely accepted that ACL rupture is a complex disorder with both genetic and environmental contributions to disease risk [7]. Given the prevalence of ACL rupture in human beings and substantial morbidity associated with the condition, identification of individuals at risk of ACL rupture before they become patients would provide the opportunity to intervene, thereby reducing the overall incidence of ACL rupture, the number of surgical procedures performed, and costs to both the hospital and patient.

ACL rupture patients are twice as likely to have a close relative with ACL rupture, when compared with individuals without ACL rupture [8]. Family members of patients with bilateral ACL rupture are also at higher risk of ACL rupture [9]. Family history of ACL rupture and a young age substantially increases risk of both ACL graft and contralateral ACL rupture [10,11]. This suggests that a substantial genetic contribution to ACL rupture exists, although heritability has not been formally estimated. Candidate gene studies have focused on ligament matrix constituents that could influence structural properties, and genes that influence extracellular matrix remodeling. Associations have been identified with a number of genes including the α1 chain of type I collagen (*COL1A1)*, the α1 chain of type V collagen (*COL5A1*) and the α1 chain of type 12 collagen (*COL12A1*) [12–15]. The *COL1A1* polymorphism is located at the binding site for the Sp1 transcription factor [12]. These associations may influence risk of other orthopaedic conditions, such as shoulder dislocation and Achilles tendon injury [12,13]. Recently, interaction between the *COL5A1* and *COL12A1* variants has been confirmed [15]. In other candidate gene studies, association between ACL rupture and polymorphisms in matrix metalloproteinase genes has been identified [16–17], particularly association with a polymorphism in the promotor region of matrix metalloproteinase-3 (*MMP-3*, stromelysin-1) in athletes [16]. Associations between polymorphisms in vascular endothelial factor A (*VEGFA*) and kinase insert-domain receptor (*KDR*), genes involved in angiogenesis signaling have also been found [18]. Some of these associations are gender-specific [13,14,18].

In addition to human genetic studies, genome-wide association study (GWAS) has been undertaken in domestic dogs (*Canis lupus familiaris*) with ACL rupture. Spontaneous naturally occurring non-contact ACL rupture is common in domestic dogs [19]. Similar to human beings, familial (breed) susceptibility and second ACL ruptures are typical [19,20]. While no animal model can perfectly mirror the biomechanics of the human knee, the canine knee joint has been established as a model for human knee pathology for several decades [21]. Dogs, like other animal models, are quadrupedal which alters weight-bearing, gait, and joint range of motion. However, the relative dimensions of the cruciate ligaments, menisci, and intercondylar notch are similar between dogs and humans. Additionally, studies have shown that, when compared to other large animal models, the canine ACL is most similar to humans with respect to cell density, blood vessel density, and cell shape [22].

The dog is also an important model organism for comparative genomic studies, because selective breeding has created distinct genetically isolated populations (breeds) with extensive linkage disequilibrium (LD) and haplotype blocks that are ~10 to 100 times longer than in humans [23,24]. Consequently, the dog is an important translational animal model for genomic one medicine research. The most important risk factor for ACL rupture in dogs is genetic background (breed) [20]. Disease prevalence exceeds 5% in several breeds (incidence of ~5,000 cases/100,000 dog years), ~100-fold higher than humans, whereas other breeds, such as the Greyhound, experience a much lower disease incidence [2,3,20]. Heritability of canine ACL rupture has been estimated at 0.27 in the Newfoundland and 0.28 in the Boxer [25,26]. Candidate gene research using SNP genotyping in dogs has also implicated genes that regulate extracellular matrix composition and ligament strength, including *COL1A1* and *COL5A1* as risk loci for the trait [27]. GWAS has shown that canine ACL rupture is a complex trait with loci on canine chromosomes (CFA) 1, 3, and 33 in the Newfoundland [28]. Association signals in the *SORCS2* and *SEMA5B* genes suggest neuronal signaling pathways may also influence risk of ACL rupture [28].

We present a GWAS in the domestic dog to discover additional candidate loci associated with ACL rupture. To take advantage of the LD structure in dogs, the GWAS was conducted in a single high-risk breed, the Labrador Retriever, the most common purebred dog in the USA. Prevalence of ACL rupture in this breed is 5.79% [20]. The GWAS identified 99 candidate loci that influence risk of ACL rupture. A SNP on CFA 24 was significantly associated with ACL rupture. Pathway analysis implicated c-type lectins, such as aggrecan, in the disease pathogenesis.

## Materials and methods

### Ethics statement

All procedures were performed in strict accordance with the recommendations in the Guide for the Care and Use of Laboratory Animals of the National Institutes of Health and the American Veterinary Medical Association and with approval from the Animal Care Committee of the University of Wisconsin-Madison (protocol #V1070). Informed consent of each owner was obtained before participation in the study.

### Canine samples and phenotyping

DNA was isolated from client-owned Labrador Retrievers using blood or saliva collection swabs. A four-generation pedigree was collected from each dog to ensure purebred status and identify siblings, which were excluded from the GWAS. Each dog underwent an orthopaedic examination that included assessment of knee stability [29]. Radiographs of the affected knee(s) were also assessed in cases. In addition, lateral weight-bearing knee radiographs were made to screen phenotype-negative control dogs. While it is not possible to identify the cruciate ligaments radiographically in the dog, compression of the infrapatellar fat pad in the knee by synovial effusion and knee osteophytosis are degenerative changes typically associated with ACL rupture [30]. Dogs were considered cases if anterior translation of the tibia relative to the femur was detected clinically and radiographic signs were consistent with ACL rupture. Labrador Retrievers ≥8 years of age have ~6% chance of developing ACL rupture [20,31]. Therefore, control dogs were ≥8 years of age with a normal orthopaedic clinical exam and normal knee radiographs.

### Genome-wide association

Genome-wide SNP genotyping was performed in 98 cases and 139 controls using the Illumina CanineHD BeadChip, which genotypes 173,662 SNPs evenly spaced across the genome. Data underwent quality control filtering using PLINK [32]. All samples had a genotyping call rate of ≥95%. 49,859 SNPs were excluded because minor allele frequency (MAF) was ≤0.05 and 7,468 SNPs were excluded because of a low genotyping rate (≤95%). 153 SNPs were excluded because of deviation from Hardy-Weinberg equilibrium at *P*<1E-07. 118,992 SNPs were used for further analysis.

To account for ancestral population structure and family relatedness in the study dogs, single marker linear mixed model (LMM) analysis was performed using GCTA (Genome-wide Complex Trait Analysis) [33] and GEMMA (Genome-wide Efficient Mixed Model Association) [34], software tools optimized for complex trait GWAS. Penalized Unified Multiple-locus Association (PUMA), in which all SNPs are analyzed together, was also used to aid detection of weaker associations often found in complex traits [35]. We used logistic regression and a 2D-MCP penalty for this analysis [36]. In the PUMA analysis, the first 20 eigenvectors were used as covariates in the association analysis to correct for population structure. Eigenvectors were obtained by principal component analysis using GCTA. Because neutering has a significant effect on risk of ACL rupture [20,37], it was included as a covariate with the GEMMA, GCTA, and PUMA analyses.

### Genome-wide significance

We defined genome-wide significance using permutation testing. Use of a Bonferroni correction for the number of SNPs tested is too conservative in dog breeds, as extensive LD means that SNPs are often inherited in haplotype blocks [24]. We defined genome-wide significance by randomly permuting the phenotypes and re-running the association analysis 1,000 times. Genome-wide significance was defined by identifying the 5% quantile of the set of minimum *P*-values from the GWAS permutations. Additionally, we estimated the number of haplotype blocks in the Labrador Retriever SNP data using PLINK [32], with LD windows of 500kb, 1Mb, and 5Mb and used the number of haplotype blocks to estimate genome-wide significance by Bonferroni correction of *P*<0.05. To facilitate further dissection of genetic variants associated with the ACL phenotype, we also identified a larger set of candidate ACL rupture regions at *P*<5E-04 [38]. Although some of the regions included may not be true associations, this would likely weaken rather than strengthen the gene set and pathway analyses, leading to false negatives rather than false positives [38].

### Defining associated loci in the genome

Linkage-disequilibrium (LD) clumping using PLINK [32] was used to define regions of association with the ACL rupture trait from the GWAS results. LD clumping defined regions around SNPs associated at *P*<5E-04 within 1 Mb of the index SNP (r^2^>0.8 and *P*<0.01). We also used GCTA to estimate in-breeding coefficient and the amount of phenotypic variance explained by the associated loci, which were defined as SNPs with r^2^>0.2 within 5Mb of the peak SNP in each locus [33,38,39].

For complex trait GWAS with a large number of risk loci, sites that are not discovered are expected to have smaller effect sizes in a second generation GWAS, because those with larger effect sizes will have been identified in the first round of GWAS [40]. To estimate the number of risk loci that are likely associated with ACL rupture, we used INPower [40]. Odds ratios were corrected for the winner’s curse before INPower analysis was performed [40,41].

### Genetic risk score computation

Two approaches were used to calculate the genetic risk scores (GRS), a simple risk alleles count method (cGRS) and a weighted method (wGRS) [42]. The wGRS weights each risk allele by the logarithm odds ratio (Log(OR)) for that allele. The wGRS is a linear combination of the number of risk alleles weighted by the Log(OR) as coefficients [42]. The Mann-Whitney U test was used to compare cGRS scores for each LMM in case and control groups. To estimate the total risk captured by the genetic risk scoring for each LMM, we calculated the odds ratios according to the wGRS quartiles [42]. We also measured the discriminative power attributable to the GRS by plotting receiver operating characteristic (ROC) curves and calculated the area under the curve (AUC) for the Labrador Retriever case and control dogs. AUC 95% confidence intervals were calculated using 2000 stratified bootstrap replicates. An R software package (pROC) was used for these analyses [43].

### Pathway analysis

Pathway analysis was performed with two methods. DAVID [44] analyses were run on the ACL rupture loci identified from our GWAS. ACL rupture loci were transposed to CanFam 3.1 coordinates (genome.ucsc.edu/cgi-bin/hgLiftOver) with 500kb flanks added to the start and end, gene size correction turned on, and stringency set to high. A list of genes from the LiftOver coordinates was then analyzed. Probability values were evaluated after Benjamini correction with DAVID.

Pathway analysis with INRICH was performed on canFam2 intervals using a map file lifted over from the canFam3.1 Broad Improved Canine Annotation catalog (UCSC Genome Browser) [45]. We used 1,000,000 permutations matched for region size, SNP density, and gene number. INRICH reports significance for each gene set and the experiment-wide significance, correcting for the number of gene sets (*P*_corr_). We considered *P*_corr_<0.05 to be significant [38]. We tested gene sets from the KEGG (Kyoto Encyclopedia of Genes and Genomes), Gene Ontology, and MSigDB (Molecular Signatures Database).

### Heritability estimation

Genomic heritability was estimated from SNPs using the BGLR statistical package [46]. SNPs with missing genotypes were filtered out using PLINK [32]. Heritability estimation was performed using 99,103 SNPs. A genomic best linear unbiased prediction (GBLUP) model was fitted using a SNP-derived genomic relationship matrix, which is equivalent to a non-parametric reproducing kernel Hilbert spaces (RKHS) method [46]. Narrow sense genetic heritability was also estimated using a data matrix prepared from pedigrees. To fit the model, 30,000 iterations of the Gibbs sampler were used with burn-in of 5,000 iterations. A correction factor was used to transform the heritability estimate on the observed scale from the regression model to the liability scale for a binary trait [47] and a population prevalence of 0.0579 [20] was used for this correction.

## Results

### GWAS population of Labrador Retrievers

**Fig 1:**
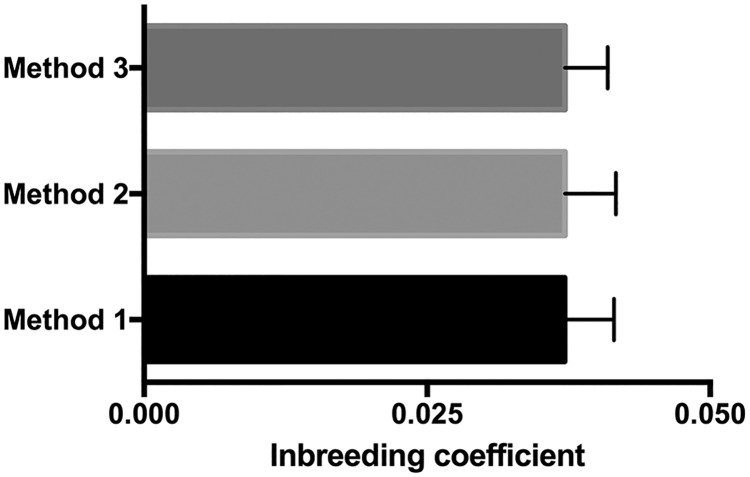
The Labrador Retriever is a dog breed with a relatively low amount of inbreeding. Whiskers represent the standard error of the mean for each analysis method (n = 237 dogs).

We genotyped 237 Labrador Retrievers using the Illumina CanineHD BeadChip. All dogs had individual call rates of >95%. The final dataset contained 118,992 SNPs from 98 cases and 139 phenotype-negative controls. Median inbreeding coefficient was 0.025 (Fig 1). The ratio of females to males in the case and control groups was 0.92 and 0.83 respectively. Of the 114 females, 99 were ovariohysterectomized (0.87). Of the 123 males, 96 were castrated (0.78). Neutered animals were distributed similarly among case and control groups (χ^2^ = 0.223, *P* = 0.6368). Mean age of the dogs in the case and control groups was 6.0±2.5 years and 10.4±1.7 years, respectively, and was significantly different (*P*<0.0001).

### GWAS identifies 99 regions associated with anterior cruciate ligament rupture

**Fig 2:**
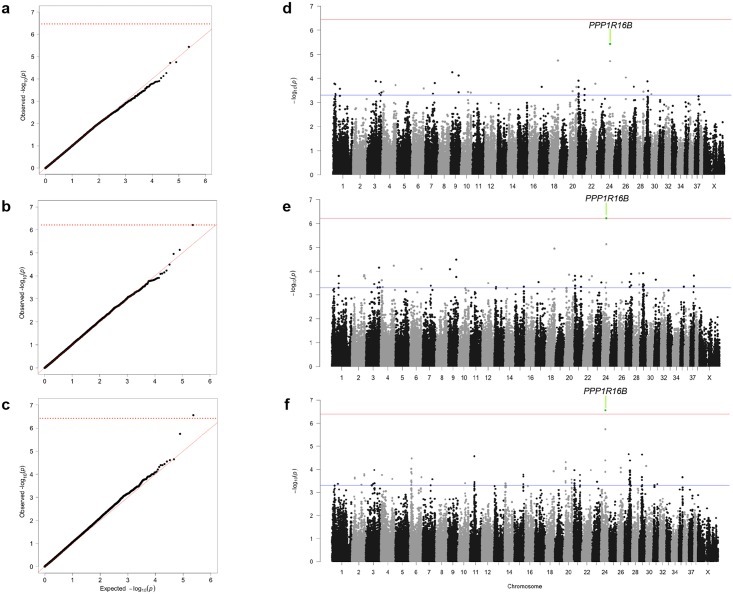
Linear mixed model GWAS corrects for population structure and identifies 99 ACL associated loci explaining a large proportion of phenotypic variance. For each linear mixed model (LMM), the QQ plots show no evidence of population stratification relative to the expected distribution. Permutation testing with each model determined genome-wide significance at (**a**) *P*<3.63E-7 for GCTA (Genome-wide Complex Trait Analysis) [33], λ = 0.987 (**b**) *P*<6.097E-7 for GEMMA (Genome-wide Efficient Mixed Model Association) [34], λ = 0.994 and (**c**) *P*<4.01E-7 for PUMA (Penalized Unified Multiple-locus Association) [35], λ = 1.012. The plots represent analysis of 118,992 SNPs from 98 cases and 139 phenotype-negative controls. (**d**) With GCTA, 36 loci have *P*<5E-4, with the most significant locus located in CFA 24, which did not meet genome-wide significance defined by minimum p-values from permutation testing. (**e**) With GEMMA, 47 loci have *P*<5E-4, with the locus on CFA 24 meeting genome-wide significance defined by minimum p-values from permutation testing. (**f**) With PUMA, 65 loci were significant at *P*<5E-4 and the locus on CFA 24 exceeded genome-wide significance defined by minimum p-values from permutation testing. The single SNP that met genome-wide significance lies within the gene *PPP1R16B*.

We tested for association between ACL rupture and SNPs with a MAF >0.05 in the Labrador Retriever breed, controlling for cryptic relatedness and population structure using linear mixed model (LMM) analysis with three approaches, including a penalized multiple regression method for improved detection of weak associations. We identified all SNPS with either significant association based on analysis of 1,000 random phenotype permutations to define genome-wide significance (*P*<1.549E-06 for GCTA, *P*<6.097E-07 for GEMMA and *P*<4.35E-07 for PUMA) or suggestive association (*P*<5E-04; Fig 2; Methods) and defined regions of association through linkage disequilibrium (Table 1, S1 Table). Control dogs were considered phenotype-negative because of the selection criteria used for recruitment. We identified 21,713, 21,754, and 21,861 haplotype blocks in the Labrador Retriever genome with LD windows of 5Mb, 1Mb, and 5kb respectively, yielding a genome-wide significance estimate of *P*<2.29E-06 to *P*<2.30E-06.

**Table 1:** ACL rupture associated loci identified by GWAS in the Labrador Retriever, a dog breed with a high disease prevalence.

SNP	Chr	Position	*P*	LMM	Risk allele	f(A)	f(U)	OR	Region start-end	Size (kb)	Genes
BICF2G630500368	24	30241088	2.76E-07	1,2,3	G	0.83	0.66	2.56	30241088–30245795	5	*BPI*, *LBP*, *RALGAPB*, *SLC32A1*, *ADIG*, *ACTR5*, *PPP1R16B*, *FAM83D*, *DHX35*
BICF2P1121006	18	54279578	1.11E-05	1,2,3	A	0.63	0.42	2.28	No LD		Many (40 genes)
BICF2S2356299	27	30557856	2.21E-05	2,3	A	0.43	0.27	2.03	No LD		*AEBP2*, *PLEKHA5*
BICF2P483191	29	21601273	2.31E-05	1,2,3	C	0.73	0.51	2.54	No LD		C29H8orf34, *SULF1*, *SLCO5A1*
BICF2P50610	11	32270617	2.75E-05	3	A	0.29	0.19	1.7	31939564–32270617	331	C11H9orf123, *PTPRD*
BICF2P890246	9	53427907	3.23E-05	1,2	A	0.16	0.36	2.99	53427907–53432248	4	Many (20 genes)
BICF2S23324965	6	14077648	3.36E-05	3	G	0.68	0.60	1.42	14077648–14092057	14	*TRRAP*, *TMEM130*, *NPTX2*, *BAIAP2L1*, *BRI3*, *TECPR1*, *LMTK2*, *PMS2*, *EIF2AK1*, *ANKRD61*, *USP42*, *CYTH3*
BICF2P544126	24	29772193	4.09E-05	3	G	0.94	0.87	2.28	29772193–29794411	22	*CTNNBL1*, *VSTM2L*, *TTI1*, *RPRD1B*, *TGM2*, *KIAA1755*, *BPI*, *LBP*, *RALGAPB*, *ADIG*, *SLC32A1*, *ACTR5*, *PPP1R16B*, *FAM83D*
BICF2P526639	27	39217437	4.12E-05	2,3	G	0.23	0.12	2.18	39211186–39217437	6	*KLRD1*, *GABARAPL1*, *TMEM52B*, *OLR1*, *CLEC7A*, *CLEC1B*, *CLEC12B*, *CLEC12A*, *CLEC2B*, *KLRF1*, *CD69*, *CLEC2D*, *KLRB1*
BICF2P1462185	20	15053718	4.89E-05	3	A	0.85	0.74	1.90	14838270–15053718	215	*EDEM1*, *ARL8B*
BICF2P1208798	9	12671217	5.49E-05	1,2	G	0.56	0.36	2.27	No LD		Many (20 genes)
BICF2G630175389	4	84260906	5.87E-05	1,2	A	0.83	0.68	2.28	No LD		*CDH10*
BICF2S24415473	3	86974042	7.07E-05	1,2	G	0.40	0.26	1.97	86948527–86974042	26	*STIM2*, *TBC1D19*, *CCKAR*, *RBPJ*, *SEL1L3*
BICF2G630412697	30	3126573	7.22E-05	1,3	G	0.96	0.86	4.23	No LD		*OR4K2*, *OR4K1*, *ORFN5*
BICF2P498515	6	75848537	7.89E-05	1,2,3	A	0.16	0.06	3.11	No LD		*LRRIQ3*
BICF2P792911	26	22894961	8.55–05	1,2,3	G	0.44	0.27	2.14	No LD		*ADRBK2*, *MYO18B*, *SEZ6L*, *ASPHD2*, *HPS4*, *SRRD*, *TFP11*, *TPST2*, *CRYBB1*, *CRBA4*
BICF2G630810143	6	11130832	9.46E-05	3	A	0.44	0.32	1.72	11130832–11177149	46	*UPK3B*, *LRWD1*, *ALKBH4*, *ORAI2*, *PRKRIP1*, *SH2B2*, *CUX1*, *MYL10*, *RABL5*, *FIS1*, *ZNHIT1*, *PLOD3*
BICF2P564273	3	55250188	1.07E-04	1,2,3	A	0.70	0.52	2.16	No LD		*ACAN*, *HAPLN3*, *MFGE8*, *ABHD2*, *RLBP1*, *FANCI*, *POLG*, *RHCG*, *TICRR*, C3H15orf38, *KIF7*, *PLIN1*, *PEX11A*, *WDR93*, *AMPN*, C3H15orf38
TIGRP2P297337	22	58201452	1.08E-04	1,2,3	A	0.44	0.27	2.2	No LD		*EFNB2*, *ARGLU1*
BICF2G630658881	21	7582214	1.09E-04	1,2,3	G	0.49	0.32	2.12	7581714–8383209	800	*JRKL*, *CCDC82*, *MAML2*, *MTMR2*, *CEP57*, *FAM76B*, *SESN3*

**Note**: OR (odds ratio) calculated from PLINK [32]. LMM Linear mixed model 1 –GCTA [33], 2 –GEMMA [34], 3 –PUMA [35]. F(A) and F(U) represent the frequency of the risk allele in case and control dogs, respectively. Data represent the twenty most significant loci of 99 associations with canine ACL rupture. SNP position and genomic regions are based on canFam2. Genes lists were derived from the SNP locus or LD block with 500kb flanking regions after liftover to canFam 3.1.

**Fig 3:**
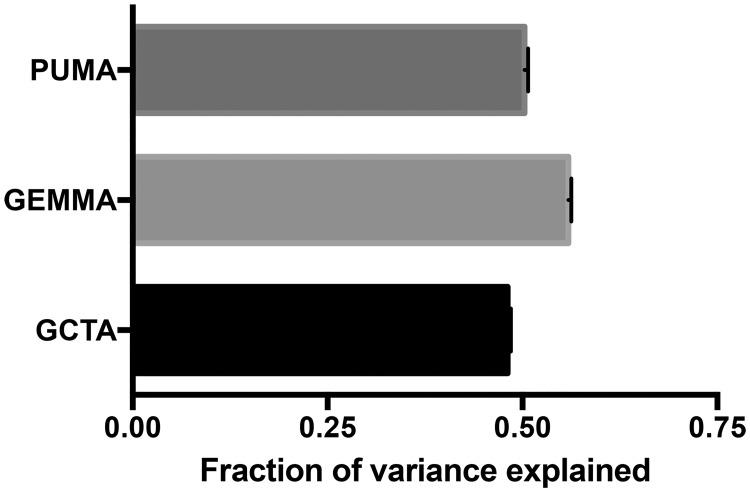
Phenotype variance was explained to a large degree by the associated genomic loci. Loci identified by linear mixed model (LMM) analysis were broadly defined as SNPs with r^2^>0.5 within 5Mb of the peak SNP. (**a**) For GCTA, 36 loci in 72.7Mb of the genome explained 48.09% of the phenotypic variance. (**b**) For GEMMA, 47 loci in 82.7Mb of the genome explained 55.88% of the phenotypic variance. (**c**) For PUMA, 65 loci in 86.58Mb of the genome explained 50.28% of the phenotypic variance in the ACL rupture trait. Whiskers represent the standard error of the mean.

With the Labrador Retriever breed, associated regions (*P*<5E-04) explained approximately half of the phenotypic variance in the ACL rupture trait (Fig 3). For GCTA, 36 loci in 72.7Mb of the genome explained 48.09% of the phenotypic variance. For GEMMA, 47 loci in 82.7Mb of the genome explained 55.88% of the phenotypic variance. For PUMA, 65 loci in 86.58Mb of the genome explained 50.28% of the phenotypic variance in the ACL rupture trait.

We identified 129 SNPs associated with canine ACL rupture. Using LD clumping, we found that these SNPs reside in 99 loci. Two of these regions were located in uncharacterized or non-coding regions of the genome. A SNP on CFA24 met genome-wide significance for LMM association analysis with GEMMA (*P* = 6.10E-07) and PUMA (*P* = 2.77E-07), but not GCTA (*P* = 3.63E-06). This SNP resides in a 5kb haplotype block with one other SNP. Nine genes are located within the locus defined by 500kb flanking regions including bactericidal/permeability-increased protein (*BPI*), lipopolysaccharide binding protein (*LBP*), Ral GTPase activation protein beta subunit (*RALGAPB*), adipogenin (*ADIG*), solute carrier family 32, member 1 (*SLC32A1*), ARP5 actin-related protein 5 (*ACTR5*), protein phosphatase 1, regulatory subunit 16B (*PPP1R16B*), family with sequence similarity 83, member D (*FAM83D*), and DEAH (Asp-Glu-Ala-His) box polypeptide 35 (*DHX35*). Although many risk loci contained large numbers of genes, two loci did not (Table 1, S1 Table), suggesting these SNPs are associated with mutations affecting gene expression (rSNPs).

Power analysis of our GWAS data set using INPower estimates that 172 loci explain the genetic contribution to ACL rupture in the Labrador Retriever (S2 Table). INPower estimates that in a future GWAS, large numbers of dogs will be needed for discovery of future loci (>1,500) (S3 Table).

### Risk loci clearly distinguish ACL rupture cases from controls

**Fig 4:**
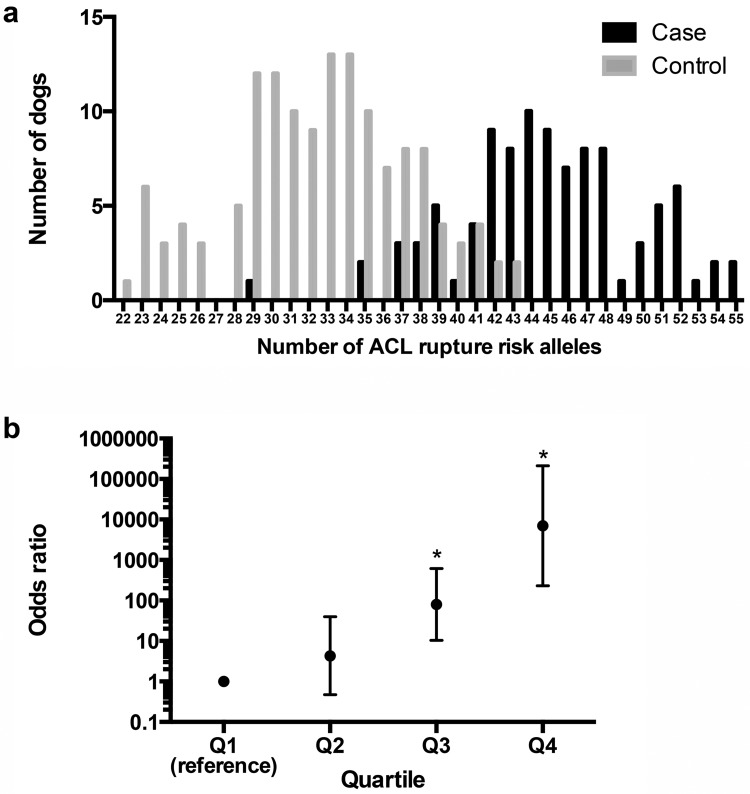
Genetic risk scoring [42] using GWAS associated loci from linear mixed model analysis with GEMMA segregates ACL rupture disease risk in case and control Labrador Retriever dogs. (**a**) Distribution of the number of ACL rupture risk loci in case and control groups of Labrador Retriever dogs. The number of risk alleles in cases and controls is significantly different (*P*<2.2E-16) (**b**) ACL rupture odds ratios of weighted genetic risk scores (wGRS) relative to the first quartile. Vertical bars represent the 95% confidence intervals. * Odds ratio is significantly different from the reference first quartile.

To evaluate the cumulative effects of associated ACL rupture risk loci, we used a genetic risk scoring approach using a simple allele count (cGRS) or a weighted approach (wGRS). We found significant differences in the number of risk alleles in cases and controls for GCTA (*P*<2.2E-16), GEMMA (*P*<2.2E-16), and PUMA (*P*<2.2E-16) (Table 2), with a shift to increased numbers of risk alleles in the cases (Fig 4). When the odds ratios according to the wGRS quartiles for each LMM were calculated, there was a significant increase in ACL rupture odds ratios with increasing wGRS quartile for all three LMM, using the first wGRS quartile as a reference (Fig 4, S1 Fig).

**Table 2:** Genetic risk scoring in ACL rupture case and control Labrador Retriever dogs using GWAS associated SNPs from linear mixed model analysis.

LMM	Number of Risk alleles		Significance
	Control	Case	
GCTA	24 (16, 40)	35 (24, 43)	*P*<2.2E-16
GEMMA	33 (22, 43)	45 (29, 55)	*P*<2.2E-16
PUMA	62 (37, 84)	77 (56, 99)	*P*<2.2E-16

**Note**: Data represent median (range) for allele counting (cGRS) [51]. LMM Linear mixed models used were GCTA [33], GEMMA [34], PUMA [35]. The Mann-Whitney U test was used to determine significance.

AUC differences between cGRS and wGRS were small and we found that there were no significant differences in ROC AUC for cGRS and wGRS for any of the three LMM analyses. For both cGRS and wGRS analyses, GCTA and GEMMA yielded increased ROC AUC values, when compared with PUMA. Overall, cGRS for GEMMA yielded the highest AUC at 0.9634, indicating that this algorithm is most efficient at classifying case and control status amongst GWAS dogs (Table 3).

**Table 3:** Receiver Operating Characteristic (ROC) analysis of genetic risk scoring in ACL rupture case and control Labrador Retriever dogs using GWAS associated SNPs from linear mixed model analysis.

LMM	cGRS		wGRS	
	AUC	95% confidence interval	AUC	95% confidence interval
GCTA	0.9487	0.9191–0.9725	0.9464	0.9183–0.9694
GEMMA	0.9634	0.9369–0.9824	0.9601	0.933–0.9801
PUMA	0.8842*	0.8356–0.9158	0.8909*	0.8458–0.9263

**Note**: LMM Linear mixed models used were GCTA [33], GEMMA [34], PUMA [35]. AUC Area under the ROC curve. Simple risk alleles count method (cGRS) and weighted method (wGRS) [42].

*Significantly different from GCTA and GEMMA (*P*<0.005 for cGRS and *P*<0.05 for wGRS).

### GWAS pathways are enriched for carbohydrate binding proteins

Functional annotation clustering using DAVID revealed significant enrichment of a cluster of 24 genes encoding carbohydrate binding proteins (*P*<1.84E-07, *P*_*corr*_ = 1.21E-04) that includes aggrecan (*ACAN*), a large structural protein that stabilizes the collagen network in ligament matrix [48]. The majority of these proteins are c-type lectin receptors (CLRs). Enrichment of a cluster of 11 genes encoding proteins with antimicrobial activity was also found (*P*<1.74E-04, *P*_*corr*_ = 0.02) (Table 4), including *LBP* and *BPI* which were also present in the locus on CFA 24 that met genome-wide significance. A third cluster of 21 genes involved with nucleosome assembly was also highly significant, but enrichment was likely overinflated as 17 of 18 genes in the cluster are located in a 1Mb region surrounding a single SNP. Using INRICH, we identified enrichment for a single set of genes (*TTR*, *SLC9A5*, *SLC10A1*, *SLC37A4*, *SLC6A1*, *AQP9*. *GABRP*, *GJB1*, *KCNJ3*, *ALB*, *GABRB3*, *P2RX1*, *SLC16A2)* (*P*<5.0E-4, *P*_*corr*_ = 0.07). This pathway primarily consists of genes encoding membrane transport proteins with a wide range of physiological functions including pH regulation, glucose homeostasis, and signal transduction.

**Table 4:** Functional annotation clustering of genes in regions associated with anterior cruciate ligament rupture identified by GWAS in the Labrador Retriever.

Cluster Term	Gene	Location start-end (CanFam 3.1)
**Carbohydrate binding**		
	*ACAN*	chr3:51995108–52032255
	*HAPLN3*	chr3:52033940–52037803
	*NPTX2*	chr6:10805532–10808481
	*CD248*	chr18:50991445–50997063
	*CLEC17A*	chr20:48069197–48079333
	*CALR*	chr20:49250753–49255568
	*MAN2B1*	chr20:49445696–49461722
	*FCER2*	chr20:52449871–52457459
	*CLEC4G*	chr20:52476065–52480126
	*CD209*	chr20:52516871–52521506
	*KLRK1*	chr27:35634936–35658993
	*KLRD1*	chr27:35698995–35706308
	*OLR1*	chr27:35828941–35838821
	*CLEC7A*	chr27:35864121–35867654
	*CLEC9A*	chr27:35919910–35934027
	*CLEC1B*	chr27:35938182–35964350
	*CLEC12B*	chr27:35953661–35961617
	*CLEC12A*	chr27:36040314–36051214
	*CLEC2B*	chr27:36120649–36145641
	*CD69*	chr27:36197203–36205042
	*CLEC2D*	chr27:36274914–36301802
	*KLRF1*	chr27:36152112–36157825
	*KLRB1*	chr27:36355936–36376701
	*KLRG1*	chr27:36740984–36742709
	*M6PR*	chr27:36784859–36798029
**Antimicrobial**		
	*DEFB132*	chr24:20589296–20592303
	*DEFB128*	chr24:20652527–20654682
	*DEFB126*	chr24:20725869–20727386
	*DEFB125*	chr24:20743523–20757512
	*DEFB118*	chr24:20771397–20900359
	*DEFB116*	chr24:20833054–20840751
	*DEFB121*	chr24:20927258–20929346
	*BPI*	chr24:26772292–26801096
	*LBP*	chr24:26810094–26821440
	*HIST1H2BG*	chr35:24178933–24179313
	*HIST1H2BC*	chr35:24989922–25000072

### ACL rupture in the Labrador Retriever has moderate heritability

Using a Bayesian method, SNP heritability and narrow sense genetic heritability of ACL rupture was estimated at 0.538 and 0.521 using SNP markers and pedigrees, respectively. After correction to the liability scale for a binary trait, these estimates were 0.493 and 0.476, respectively.

## Discussion

By undertaking a within-breed GWAS in the Labrador Retriever, we found 99 regions of association with the trait, suggesting that ACL rupture is a complex, potentially highly polygenic condition. These loci explained between 48% and 56% of the disease phenotypic variation, depending on which LMM was used for the association analysis, suggesting that inherited factors make an important contribution to the disease in the Labrador Retriever dog model. We estimated narrow sense genomic heritability to be 0.48–0.49, higher values than past estimates in the Newfoundland and Boxer breeds [25,26].

Our studied sample of Labrador Retriever dogs represented typical features of the general population, with an approximately equal number of male and female dogs and a large majority of the dogs being neutered by castration or ovariohysterectomy, respectively. ACL rupture in dogs is an acquired condition [19,30]. In the present study, ACL rupture cases were middle-aged dogs typically, with a mean age of 6.0 years. In dogs, loss of sex steroids through neutering is a risk factor for ACL rupture [20,37]. In human beings, ACL rupture is predisposed to female athletes [6]. Knee laxity in women is lowest in the follicular phase of the menstrual cycle (low estrogen), when ACL rupture is most common [49,50]. This suggests that the influence of sex steroid levels on ACL laxity in both species may influence accumulation of matrix damage over time and, consequently, risk of rupture.

A large majority of the risk loci discovered in this study differ from a recent GWAS in the Newfoundland breed, which identified loci predominantly on CFA 1, 3, and 33 [28]. Although overlap of risk loci between dog breeds needs more investigation, a different genetic architecture in different breeds suggests that this complex trait likely consists of many genetic variants that are concentrated differently in different breeds through population bottlenecks and intense selection [51]. A similar scenario likely summarizes the genetic basis of human ACL rupture [52], although GWAS of human ACL rupture has not been performed to date.

Because of the high LD within breeds of dog, risk loci often contained large numbers of genes. However, two risk loci appeared to contain rSNPs located in gene deserts in intergenic regions of the genome of >500kb that lack annotated genes or protein-coding sequences [53]. Complex trait disease is caused by disturbance to biological networks, not by isolated genes or proteins. Regulatory SNPs can influence gene expression through a number of mechanisms that include the three dimensional organization of the genome, RNA splicing, transcription factor binding, DNA methylation, and long non-coding RNAs (lncRNA) [53,54]. Investigation of SNPs associated with complex trait disease in dogs with potential regulatory function through expressed quantitative trait loci (eQTL) studies or other methods [54] is currently lacking.

One locus consisting of a 5kb haplotype block with one other SNP on CFA 24 met genome-wide significance in the present study. Nine genes were identified in this block with diverse physiological effects on cellular and tissue homeostasis. For example, *ACTR5* plays an important role in chromatin remodeling during transcription, DNA repair, and DNA regulation [55]. *DHX35* encodes an ATP-ase that plays a role in RNA splicing [56]. *RALBAPB* as well as *FAM83D* are both important for mitotic regulation [57,58]. While a relationship between cellular homeostasis/proliferation and ACL rupture has not been established, it is feasible that aberrations in the genes that govern these processes could have a wide range of effects that may alter ligament tissue integrity and homeostasis. Other genes in this block include *LBP* and *BPI*, which have an important function regarding immuno-stimulatory capacity of innate immune mechanisms. Notably, our top SNP resides within *PPP1R16B*. *PPP1R16B* encodes a protein that promotes angiogenesis through inhibition of phosphatase and tensin homolog (*PTEN*) [59]. Angiogenesis-associated signaling is important for ligament matrix remodeling after mechanical loading, and variations in this cascade have been associated with non-contact ACL rupture risk in human beings [18].

To further investigate the large number of genes we identified within risk loci, we also undertook pathway analysis of our data using two different methods. Pathway analysis using DAVID revealed an association with a cluster of 24 carbohydrate-binding protein genes. The majority of these proteins were CLRs. CLRs primarily function as pattern recognition receptors; they play roles in activation or suppression of the immune response through recognition of microbial, fungal, or self molecules, including recognition of MHC class 1 [60]. Aberrant immune function may play a role in the pathogenesis of canine ACL rupture, as development of synovial inflammation is an early event in disease pathogenesis [61] and is a significant factor influencing disease progression [30]. Other genes in the cluster include aggrecan (*ACAN*) and hyaluronan and proteoglycan link protein 3 (*HAPLN3*), or cartilage link protein. Aggrecan is a large aggregating proteoglycan that interacts with cartilage link protein and hyaluronic acid to form stable aggregates in collagenous tissues [62]. Through binding to fixed charged groups, the proteoglycan aggregate maintains osmotic pressure in collagenous tissues to promote water retention. Tissue hydration is important for efficient distribution of load and for the ability of cells to accomplish repair [63]. Equine degenerative suspensory ligament desmitis (DSLD), a debilitating disorder of horses that leads to collagen disruption and eventual rupture of the suspensory ligament, is associated with a 15-fold increase in aggrecan content of affected ligaments [64]. Moreover, recent work has linked human *ACAN* rs1516797 with the risk of ACL injury in both males and females [48]. A separate study revealed *ACAN* gene expression is up-regulated in ACL samples from female compared to male patients that have undergone ACL repair surgery, suggesting a possible etiology for the observed sex differences among patients with ACL injury [65]. The precise mechanism by which *ACAN* up-regulation may lead to ligament weakening is currently unclear, though a structural change appears to be the most likely etiology [63,64].

DAVID also revealed an association with a cluster of 11 genes encoding proteins with antimicrobial activity, including *LBP* and *BPI*, which reside within the locus that met genome-wide significance in this study. These proteins work together to bind lipopolysaccharide, aiding in host defense against gram-negative organisms. DNA from a wide variety of bacterial species has been identified in the synovium of both human and canine arthritis patients [66,67]. While a causal link between the presence of bacterial DNA and development of arthritis has not yet been established, this information suggests that interactions between antimicrobial proteins and environmental bacteria may play a role in the pathogenesis of ACL rupture. Additionally, seven of the genes in this cluster encode beta-defensin proteins. Defensins are antibiotic peptides involved in host defense at epithelial and mesenchymal surfaces. Increased expression of beta defensins has been implicated in the pathogenesis of osteoarthritis in both human beings and mouse models [68].

We also tested genomic regions associated with ACL rupture for gene set enrichment using INRICH. One pathway, module 415 from the Molecular Signatures Database, was inflated. This pathway included 13 genes, most of which encode membrane transport proteins with various physiological roles. *GJBI* is a member of the large connexin family and encodes connexin 32, a gap junction protein that has been implicated in the regulation of collagen synthesis and the matrix remodeling response to mechanical loading of tendon [69,70]. Other genes in this module are associated with central nervous system function. *SLC6A1*, *GABRP*, and *GABRB3* are all associated with GABA signaling and mutations in TTR and have been associated with sensorimotor polyneuropathy [71]. Previous work has suggested a role for neurological pathways in susceptibility to ACL rupture in Newfoundland dogs [28].

ACL rupture GRSs were calculated for each dog to determine the cumulative effect of ACL rupture-associated loci on disease risk. While previous work found that wGRS better accounted for genetic risk [42], our study found no difference between cGRS and wGRS for any of the LMMs used. This is consistent with the idea that the ACL rupture phenotype is associated with a large number of genetic loci with small effects. In diseases with genetic loci with large effects, wGRS would more accurately represent the cumulative effect of individual loci on genetic risk. Overall, classification capability of GRS is high, with a cGRS for GEMMA AUC of approximately 96%, indicating that we have clearly captured genetic loci that contribute to ACL rupture risk in our LMM association analysis. Additionally, the proportion of variance explained by risk SNPs was calculated separately for each algorithm. Of the three, SNPs identified by GEMMA captured the largest proportion of variance. It should be noted that these estimates for classification capability as well as phenotypic variance explained are likely inflated with regard to genomic prediction as the same data were used for SNP selection, classification capability, and variance estimation. Future work should include investigation of predictive ability by applying these methods to an independent test cohort of case and control dogs.

SNP-based and pedigree-based heritability of ACL rupture were estimated at 0.49 and 0.48, respectively, using a Bayesian method. These estimates are considerably higher than restricted maximum likelihood (REML) heritability estimates calculated for other breeds of dog [25,26]. It is unclear whether ACL rupture is truly more heritable in the Labrador Retriever than in other breeds or if the higher value is a reflection of sampling variation or the Bayesian method used. REML estimation of heritability was attempted but the algorithm did not converge, probably because of the size or structure of the data set.

A limitation for this study is sample size. Canine GWAS for a complex trait requires approximately 100 cases and 100 controls to detect a five-fold risk allele [51], suggesting our study has reasonable power. Only one of 99 regions identified as associated with ACL rupture met genome-wide significance. For complex trait diseases in dogs with high population prevalence and risk alleles that have relatively small effects, larger data sets with additional dogs may be required to improve statistical power. INPower analysis of our data suggested that >1500 dogs may be required to capture additional loci that contribute to the ACL rupture phenotype.

In conclusion, detailed analysis of the genetic risk factors involved in the initiation and progression of ACL rupture will provide a clear understanding of the genetic factors that cause disturbance to biological networks sufficient to lead to ACL rupture. Our data suggest that genetic risk of ACL rupture is influenced by multiple genomic loci with small individual additive effects. In our association study, we clearly show that ACL rupture is a highly polygenic trait. Our results suggest that biological networks that control innate immune mechanisms, aggrecan (*ACAN*) signaling, cellular proliferation, membrane transport, and/or neuronal signaling pathways should be further investigated. The genetic loci we have identified in this study will guide further dissection of genomic variants associated with the ACL rupture phenotype. Importantly, dogs have long been studied as genetic models of human disease. Here, we highlight how dogs are a genetically amenable model organism for studying orthopaedic complex trait disease and provide a model system that facilitates complex trait dissection. Genetic variants that contribute to disease risk in dogs may be investigated for their potential affect in human populations. Ultimately, insights gained from this research may also lead to novel treatments and advances in complex trait genomic prediction. An accurate genomic prediction algorithm for ACL rupture in both human beings and dogs could be used to identify at-risk subjects before rupture occurs. This information could be used to control environmental variables that are known to contribute to ACL rupture risk. It would also provide the opportunity for medical intervention. Such advances could have a large impact on human and animal health.

## Supporting information

S1 Fig Genetic risk scoring using GWAS associated loci from linear mixed model analysis with GCTA [33], GEMMA [34], and PUMA [35] segregates ACL rupture disease risk in case and control Labrador Retriever dogs. (PDF)

S1 Table Anterior cruciate ligament rupture associated SNPs identified by GWAS in the Labrador Retriever, a dog breed with a high disease prevalence. (PDF)

S2 Table Statistical power and odds ratio correction for anterior cruciate ligament rupture GWAS risk loci identified by GEMMA in the Labrador Retriever. (PDF)

S3 Table Estimation of the expected number of loci to be discovered in a future GWAS of ACL rupture in the Labrador Retriever using INPower. (PDF)

## References

[1] von Porat A, Roos EM, Roos H. High prevalence of osteoarthritis 14 years after an anterior cruciate ligament tear in male soccer players: A study of radiographic and patient relevant outcomes. Ann Rheum Dis. 2004;63: 269–273. 10.1136/ard.2003.008136 14962961 PMC1754918

[2] Miyasaka KC, Daniel DM, Stone ML, Hirshman P. The incidence of knee ligament injuries in the general population. Am J Knee Surg. 1991;4: 3–8.

[3] Gianotti SM, Marshall SW, Hume PA, Bunt L. Incidence of anterior cruciate ligament injury and other knee ligament injuries: A national population-based study. J Sci Med Sport. 2009;12: 622–627. 10.1016/j.jsams.2008.07.005 18835221

[4] Griffin LY, Agel J, Albohm MJ, Arendt EA, Dick RW, Garrett WE, et al Noncontact anterior cruciate ligament injuries: Risk factors and prevention strategies. J Am Acad Orthop Surg. 2000;8: 141 10874221 10.5435/00124635-200005000-00001

[5] Wright RW, Magnussen RA, Dunn WR, Spindler KP. Ipsilateral graft and contralateral ACL rupture at five years or more following ACL reconstruction. J Bone Joint Surg Am. 2011;93: 1159–1165. 10.2106/JBJS.J.00898 21776554 PMC3110421

[6] Sutton KM, Bullock JM. Anterior cruciate ligament rupture: Differences between males and females. J Am Acad Orthop Surg. 2013;21: 41–50. 10.5435/JAAOS-21-01-41 23281470

[7] September AV, Schwellnus MP, Collins M. Tendon and ligament injuries: the genetic component. Br J Sports Med. 2007;41: 241–246. 10.1136/bjsm.2006.033035 17261551 PMC2658952

[8] Flynn RK, Pedersen CL, Birmingham TB, Kirkley A, Jackowski D, Fowler PJ. The familial predisposition toward tearing the anterior cruciate ligament: A case control study. Am J Sports Med. 2005;33: 23–28. 10.1177/0363546504265678 15610995

[9] Harner CD, Paulos LE, Geenwald AE, Rosenberg TD, Cooley VC. Detailed analysis of patients with bilateral anterior cruciate ligament injuries. Am J Sports Med. 1994;22: 37–43. 10.1177/036354659402200107 8129108

[10] Brophy RH, Schmitz L, Wright RW, Dunn WR, Parker RD, Andrish JT, et al Return to play and future ACL injury risk after ACL reconstruction in soccer athletes from the Multicenter Orthopaedic Outcomes Network (MOON) group. Am J Sports Med. 2012;40: 2517–2522. 10.1177/0363546512459476 23002201 PMC3692367

[11] Webster KE, Feller JA, Leigh WB, Richmond AK. Younger patients are at increased risk for graft rupture and contralateral injury after anterior cruciate ligament reconstruction. Am J Sports Med. 2014;42: 641–647. 10.1177/0363546513517540 24451111

[12] Khoschnau S, Melhus H, Jacobson A, Rahme H, Bengtsson H, Ribom E, et al Type I collagen alpha1 Sp1 polymorphism and the risk of cruciate ligament ruptures or shoulder dislocations. Am J Sports Med. 2008;36: 2432–2436. 10.1177/0363546508320805 18669982

[13] Posthumus M, September AV, O’Cuinneagain D, van der Merwe W, Schwellnus MP, Collins M. The COL5A1 gene is associated with increased risk of anterior cruciate ligament ruptures in female participants. Am J Sports Med. 2009;37: 2234–2240. 10.1177/0363546509338266 19654427

[14] Posthumus M, September AV, O'Cuinneagain D, van der Merwe W, Schwellnus MP, Collins M. The association between the COL12A1 gene and anterior cruciate ligament ruptures. Br J Sports Med. 2009;44: 1160–1165. 10.1136/bjsm.2009.060756 19443461

[15] O'Connell K, Knight H, Ficek K, Leonska-Duniec A, Maciejewska-Karlowska A, Sawczuk M, et al Interactions between collagen gene variants and risk of anterior cruciate ligament rupture. Eur J Sport Sci. 2015;15: 341–350. 10.1080/17461391.2014.936324 25073002

[16] Malila S, Yuktanandana P, Saowaprut S. Association between matrix metalloproteinase-3 polymorphism and anterior cruciate ligament ruptures. Genet Mol Res. 2011;10: 4158–4165. 10.4238/2011.October.31.1 22057989

[17] Posthumus M, Collins M, van der Merwe L, O'Cuinneagain D, van der Merwe W, Ribbans WJ, et al Matrix metalloproteinase genes on chromosome 11q22 and the risk of anterior cruciate ligament (ACL) rupture. Scand J Med Sci Sports. 2012;22: 523–533. 10.1111/j.1600-0838.2010.01270.x 21410539

[18] Rahim M, Gibbon A, Hobbs H, van der Merwe W, Posthumus M, Collins M, et al The association of genes involved in the angiogenesis-associated signaling pathway with risk of anterior cruciate ligament rupture. J Orthop Res. 2014;32: 1612–1618. 10.1002/jor.22705 25111568

[19] Muir P, Schwartz Z, Malek S, Kreines A, Cabrera SY, Buote NJ, et al Contralateral cruciate survival in dogs with unilateral non-contact cranial cruciate ligament rupture. PLoS One. 2011;6: e25331 10.1371/journal.pone.0025331 21998650 PMC3187768

[20] Witsberger TH, Villamil JA, Schultz LG, Hahn AW, Cook JL. Prevalence of and risk factors for hip dysplasia and cranial cruciate ligament deficiency in dogs. J Am Vet Med Assoc. 2008;232: 1818–1824. 10.2460/javma.232.12.1818 18598150

[21] Gregory MH, Capito N, Kuroki K, Stoker AM, Cook JL, Sherman SL. A review of translational animal models for knee osteoarthritis. Arthritis. 2012; 764621 10.1155/2012/764621 23326663 PMC3541554

[22] Proffen BL, Murray MM. In vivo models of ACL injury (central defect, porcine, ovine, canine) In: Murray MM, Vavken P, Fleming BC, editors. The ACL handbook. New York: Springer; 2013.

[23] Sutter NB. Extensive and breed-specific linkage disequilibrium in Canis familiaris. Genome Res. 2004;14: 2388–2396. 10.1101/gr.3147604 15545498 PMC534662

[24] Lindblad-Toh K, Wade CM, Mikkelsen TS, Karlsson EK, Jaffe DB, Kamal M, et al Genome sequence, comparative analysis and haplotype structure of the domestic dog. Nature. 2005;438: 803–819. 10.1038/nature04338 16341006

[25] Wilke VL, Conzemius MG, Kinghorn BP, Macrossan PE, Cai W, Rothschild MF. Inheritance of rupture of the cranial cruciate ligament in Newfoundlands. J Am Vet Med 2006;228: 61–64. 10.2460/javma.228.1.61 16426167

[26] Nielen ALJ, Janss LLG, Knol BW. Heritability estimations for diseases, coat color, body weight, and height in a birth cohort of Boxers. Am J Vet Res. 2001;62: 1198–1206. 11497438 10.2460/ajvr.2001.62.1198

[27] Baird AEG, Carter SD, Innes JF, Ollier WE, Short AD. Genetic basis of cranial cruciate ligament rupture (CCLR) in dogs. Connect Tissue Res. 2014;55: 275–281. 10.3109/03008207.2014.910199 24684544

[28] Baird AEG, Carter SD, Innes JF, Ollier W, Short A. Genome-wide association study identifies genomic regions of association for cruciate ligament rupture in Newfoundland dogs. Anim Genet. 2014;45: 542–549. 10.1111/age.12162 24835129

[29] Muir P. Physical examination of lame dogs. Compend Contin Educ Pract Vet. 1997;19: 1149–1161.

[30] Chuang C, Ramaker MA, Kaur S, Csomos RA, Kroner KT, Bleedorn JA, et al Radiographic risk factors for contralateral rupture in dogs with unilateral cranial cruciate ligament rupture. PLoS One. 2014;9: e106389 10.1371/journal.pone.0106389 25254499 PMC4177807

[31] Reif U, Probst CW. Comparison of tibial plateau angles in normal and cranial cruciate deficient stifles of Labrador Retrievers. Vet Surg. 2004;32: 385–389. 10.1053/jvet.2003.50047 12866002

[32] Chang CC, Chow CC, Tellier LC, Vattikuti S, Purcell SM, Lee JJ. Second-generation PLINK: Rising to the challenge of larger and richer datasets. Gigascience. 2015;4: 7 10.1186/s13742-015-0047-8 25722852 PMC4342193

[33] Yang J, Lee SH, Goddard ME, Visscher PM. GCTA: A tool for genome-wide complex trait analysis. An J Hum Genet. 2011;88: 76–82. 10.1016/j.ajhg.2010.11.011 PMC3014363 21167468

[34] Zhou X, Stephens M. Genome-wide efficient mixed-model analysis for association studies. Nature Genet. 2012;44: 821–824. 10.1038/ng.2310 22706312 PMC3386377

[35] Hoffman GE, Logsdon BA, Mezey JG. PUMA: A unified framework for penalized multiple regression analysis of GWAS data. PLoS Comput Biol. 2013;9: e1003101 10.1371/journal.pcbi.1003101 23825936 PMC3694815

[36] Zhang CH. Nearly unbiased variable selection under minimax concave penalty. Ann Stat. 2010;38: 894–942

[37] Whitehair JG, Vasseur PB, Willits NH. Epidemiology of cranial cruciate ligament rupture in dogs. J Am Vet Med Assoc. 1993;203: 1016–1019. 8226247

[38] Karlsson EK, Sigurdsson S, Ivansson E, Thomas R, Elvers I, Wright J, et al Genome-wide analyses implicate 33 loci in heritable dog osteosarcoma, including regulatory variants near CDKN2A/B. Genome Biol. 2013;14: R132 10.1186/gb-2013-14-12-r132 24330828 PMC4053774

[39] Tang R, Noh HJ, Wang D, Sigurdsson S, Swofford R, Perloski M, et al Candidate genes and functional noncoding variants identified in a canine model of obsessive-compulsive disorder. Genome Biol. 2014;15: R25 10.1186/gb-2014-15-3-r25 24995881 PMC4038740

[40] Park J-H, Wacholder S, Gail MH, Peters U, Jacobs KB, Chanock SJ, et al Estimation of effect size distribution from genome-wide association studies and implications for future discoveries. Nature Genet. 2010;42: 570–575. 10.1038/ng.610 20562874 PMC4615599

[41] Ghosh A, Zou F, Wright FA. Estimating odds ratios in genome scans: An approximate conditional likelihood approach. Am J Hum Genet. 2008;82: 1064–1074. 10.1016/j.ajhg.2008.03.002 18423522 PMC2665019

[42] Chen H, Poon A, Yeung C, Helms C, Pons J, Bowcock AM, et al A genetic risk score combining ten psoriasis risk loci improves disease prediction. PLoS One. 2011;6: e19454 10.1371/journal.pone.0019454 21559375 PMC3084857

[43] Robin X, Turck N, Hainard A, Tiberti N, Lisacek F, Sanchez JC, Müller M. pROC: An open-source package for R and S+ to analyze and compare ROC curves. BMC Bioinformatics. 2011;12: 77 10.1186/1471-2105-12-77 21414208 PMC3068975

[44] Huang DW, Sherman BT, Lempicki RA. Systematic and integrative analysis of large gene lists using DAVID bioinformatics resources. Nature Protoc. 2009;4: 44–57. 19131956 10.1038/nprot.2008.211

[45] Lee PH, O'Dushlaine C, Thomas B, Purcell SM. INRICH: Interval-based enrichment analysis for genome-wide association studies. Bioinformatics. 2012;28: 1797–1799. 10.1093/bioinformatics/bts191 22513993 PMC3381960

[46] Pérez P, de los Campos G. Genome-wide regression and prediction with the BGLR statistical package. Genetics. 2014;198: 483–495. 10.1534/genetics.114.164442 25009151 PMC4196607

[47] Zhou X, Carbonetto P, Stephens M. Polygenic modeling with Bayesian sparse linear mixed models. PLoS Genet. 2013;9: e1003264 10.1371/journal.pgen.1003264 23408905 PMC3567190

[48] Mannion S, Mtintsilana A, Posthumus M, van der Merwe W, Hobbs H, Collins M, et al Genes encoding proteoglycans are associated with the risk of anterior cruciate ligament ruptures. Br J Sports Med. 2014;48: 1640–1646. 10.1136/bjsports-2013-093201 24552666

[49] Beynnon BD, Johnson RJ, Braun S, Sargent M, Bernstein IM, Skelly JM, et al The relationship between menstrual cycle phase and anterior cruciate ligament injury: A case-control study of recreational alpine skiers. Am J Sports Med. 2006;34: 757–764. 10.1177/0363546505282624 16436538

[50] Hewett TE, Zazulak BT, Myer GD. Effects of the menstrual cycle on anterior cruciate ligament injury risk: a systematic review. Am J Sports Med. 2007;35: 659–668. 10.1177/0363546506295699 17293469

[51] Karlsson EK, Lindblad-Toh K. Leader of the pack: Gene mapping in dogs and other model organisms. Nat Rev Genet. 2008;9: 713–725. 10.1038/nrg2382 18714291

[52] Robinson MR, Wray NR, Visscher PM. Explaining additional genetic variation in complex traits. Trends Genet. 2014;30: 124–132. 10.1016/j.tig.2014.02.003 24629526 PMC4639398

[53] Schierding W, Cutfield WS, O'Sullivan JM. The missing story behind genome wide association studies: Single nucleotide polymorphisms in gene deserts have a story to tell. Front Genet. 2014; 5: 39 10.3389/fgene.2014.00039 24600475 PMC3927098

[54] Huang Q. Genetic study of complex diseases in the post-GWAS era. J Genet Genomics. 2015;42: 87–98. 10.1016/j.jgg.2015.02.001 25819085

[55] Kitayama K, Kamo M, Oma Y, Matsuda R, Uchida T, Ikura T, et al The human actin-related protein hArp5: Nucleo-cytoplasmic shuttling and involvement in DNA repair. Exp Cell Res. 2009;315: 206–217. 10.1016/j.yexcr.2008.10.028 19014934

[56] Ilagan JO, Chalkley RJ, Burlingame AL, Jurica MS. Rearrangements within human spliceosomes captured after exon ligation. RNA. 2013;19: 400–412. 10.1261/rna.034223.112 23345524 PMC3677250

[57] Personnic N, Lakisic G, Gouin E, Rousseau A, Gautreau A, Cossart P, et al A role for Ral GTPase-activating protein subunit β in mitotic regulation. FEBS J. 2014;281: 2977–2989. 10.1111/febs.12836 24814574

[58] Liao W, Liu W, Liu X, Yuan Q, Ou Y, Qi Y, et al Upregulation of FAM83D affects the proliferation and invasion of hepatocellular carcinoma. Oncotarget. 2015;6: 24132–24147. 10.18632/oncotarget.4432 26125229 PMC4695175

[59] Obeidat M, Li L, Ballermann BJ. TIMAP promotes angiogenesis by suppressing PTEN-mediated Akt inhibition in human glomerular endothelial cells. Am J Physiol Renal Physiol. 2014;307: F623–633. 10.1152/ajprenal.00070.2014 25007873

[60] Dambuza IM, Brown GD. C-type lectins in immunity: Recent developments. Curr Opin Immunol. 2015;32: 21–27. 10.1016/j.coi.2014.12.002 25553393 PMC4589735

[61] Bleedorn JA, Greuel EN, Manley PA, Schaefer SL, Markel MD, Holzman G, et al Synovitis in dogs with stable stifle joints and incipient cranial cruciate ligament rupture: A cross-sectional study. Vet Surg. 2011;40: 531–543. 10.1111/j.1532-950X.2011.00841.x 21615432

[62] Spicer AP, Joo A, Bowling RA Jr. A hyaluronan binding link protein gene family whose members are physically linked adjacent to chrondroitin sulfate proteoglycan core protein genes. J Biol Chem. 2003;278: 21083–21091. 10.1074/jbc.M213100200 12663660

[63] Halper J. Proteoglycans and diseases of soft tissues In: Halper J, editor. Progress in heritable soft connective tissue diseases. Dordrecht: Springer Netherlands; 2014 pp 49–58.

[64] Plaas A, Sandy JD, Liu H, Diaz MA, Schenkman D, Magnus RP, et al Biochemical identification and immunolocalizaton of aggrecan, ADAMTS5 and inter-alpha-trypsin-inhibitor in equine degenerative suspensory ligament desmitis. J Orthop Res. 2011;29: 900–906. 10.1002/jor.21332 21246622

[65] Johnson JS, Morscher MA, Jones KC, Moen SM, Klonk CJ, Jacquet R, et al Gene expression differences between ruptured anterior cruciate ligaments in young male and female subjects. J Bone Joint Surg Am. 2015;97: 71–79. 10.2106/JBJS.N.00246 25568397

[66] Gérard HC, Wang Z, Wang GF, El-Gabalawy H, Goldbach-Mansky R, Li Y, et al Chromosomal DNA from a variety of bacterial species is present in synovial tissue from patients with various forms of arthritis. Arthritis Rheum. 2001;44: 1689–1697. 10.1002/1529-0131(200107)44:7<1689::AID-ART293>3.0.CO;2-K 11465721

[67] Muir P, Oldenhoff WE, Hudson AP, Manley PA, Schaefer SL, Markel MD, et al Detection of DNA from a range of bacterial species in the knee joints of dogs with inflammatory knee arthritis and associated degenerative anterior cruciate ligament rupture. Microb Pathog. 2007;42: 47–55. 10.1016/j.micpath.2006.10.002 17320342

[68] Varoga D, Paulsen FP, Kohrs S, Grohmann S, Lippross S, Mentlein R, et al Expression and regulation of human beta-defensin-2 in osteoarthritic cartilage. J Pathol. 2006;209: 166–173. 10.1002/path.1974 16622898

[69] Young NJ, Becker DL, Fleck RA, Goodship AE, Patterson-Kane JC. Maturational alterations in gap junction expression and associated collagen synthesis in response to tendon function. Matrix Biol. 2009;28: 311–323. 10.1016/j.matbio.2009.05.002 19481603

[70] Waggett AD, Benjamin M, Ralphs JR. Connexin 32 and 43 gap junctions differentially modulate tenocyte response to cyclic mechanical load. Eur J Cell Biol. 2006;85: 1145–1154. 10.1016/j.ejcb.2006.06.002 16859807

[71] Dohrn MF, Röcken C, Bleecker JL, Martin J-J, Vorgerd M, Bergh PY, et al Diagnostic hallmarks and pitfalls in late-onset progressive transthyretin-related amyloid-neuropathy. J Neurol. 2013;260: 3093–3108. 10.1007/s00415-013-7124-7 24101130

