# Supplementary material for: Genome-wide association analysis in dogs implicates 99 loci as risk variants for anterior cruciate ligament rupture
Source: PLoS One. 2017 Apr 5;12(4):e0173810. doi: 10.1371/journal.pone.0173810 (PMC5381864; doi:10.1371/journal.pone.0173810)
Supplement: S1 Fig — (PDF) [file pone.0173810.s001.pdf]

Figure S1 | Genetic risk scoring using GWAS associated loci from linear mixed model analysis with GCTA [33], GEMMA [34], and PUMA [35] segregates ACL rupture disease risk in case and control Labrador Retriever dogs. Distribution of the number of ACL rupture risk loci in case and control groups of Labrador Retriever dogs for GCTA (**a**), GEMMA (**b**), and PUMA (**c**). The number of risk alleles in cases and controls is significantly different (*P*<2.2E-16). ACL rupture odds ratios of weighted genetic risk scores (wGRS) relative to the first quartile for GCTA (**d**), GEMMA (**e**), and PUMA (**f**). Vertical bars represent the 95% confidence intervals. * Odds ratio is significantly different from the reference first quartile.

a

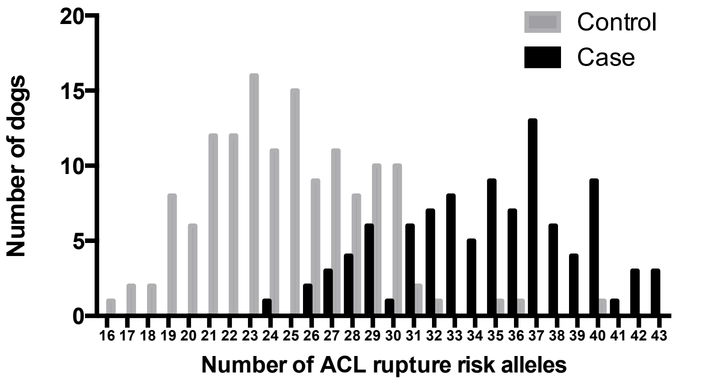

b

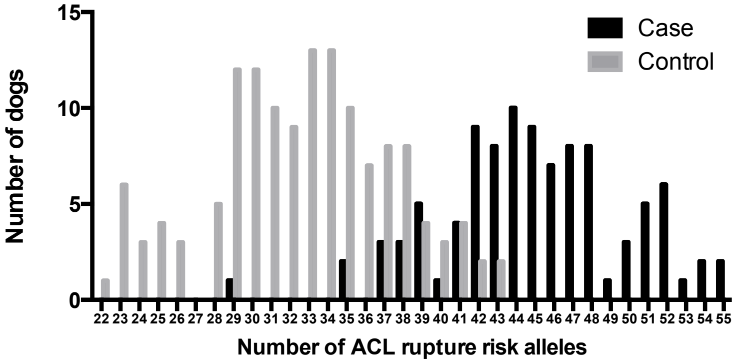

c

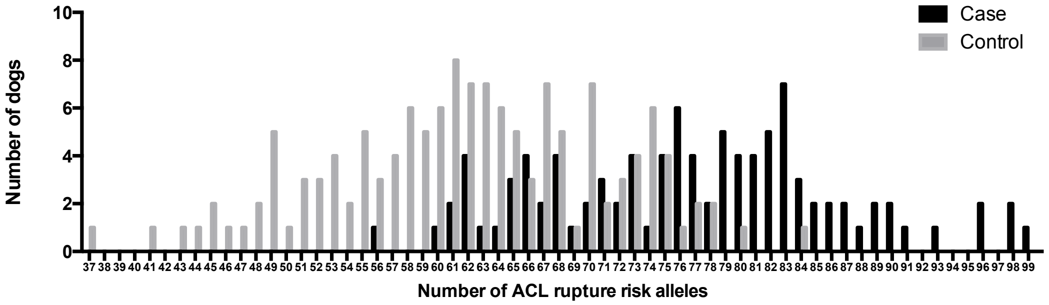
Case
Control
Number of dogs
0
2
4
6
8
10
37 38 39 40 41 42 43 44 45 46 47 48 49 50 51 52 53 54 55 56 57 58 59 60 61 62 63 64 65 66 67 68 69 70 71 72 73 74 75 76 77 78 79 80 81 82 83 84 85 86 87 88 89 90 91 92 93 94 95 96 97 98 99
Number of ACL rupture risk alleles

d

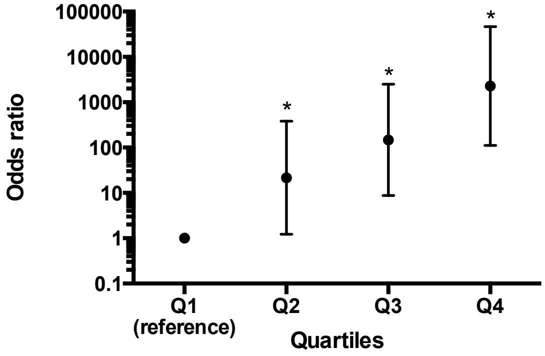
Odds ratio
100000
10000
1000
100
10
1
0.1
*
*
*
Q1
(reference)
Q2
Q3
Q4
Quartiles

e

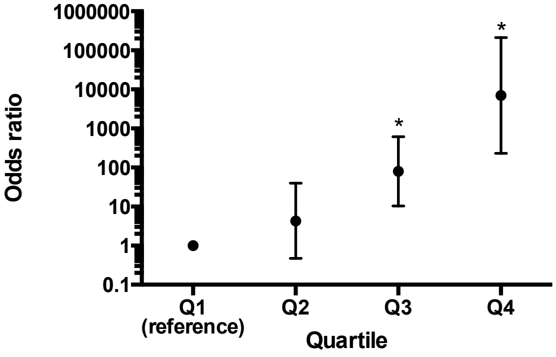
Odds ratio
1000000
100000
10000
1000
100
10
1
0.1
*
*
Q1
(reference)
Q2
Q3
Q4
Quartile

f

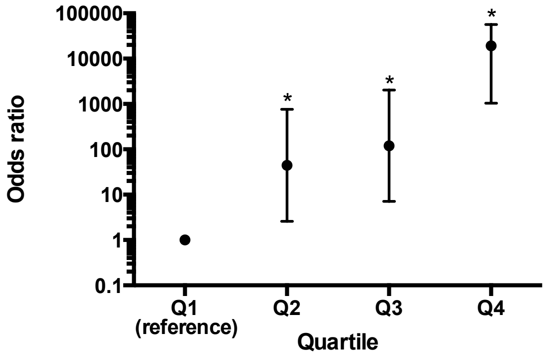
Odds ratio
100000
10000
1000
100
10
1
0.1
*
*
*
Q1
(reference)
Q2
Q3
Q4
Quartile
