# Supplementary material for: Genome-wide association analysis in dogs implicates 99 loci as risk variants for anterior cruciate ligament rupture
Source: PLoS One. 2017 Apr 5;12(4):e0173810. doi: 10.1371/journal.pone.0173810 (PMC5381864; doi:10.1371/journal.pone.0173810)
Supplement: S1 Table — (PDF) [file pone.0173810.s002.pdf]

Table S1 | Anterior cruciate ligament rupture associated SNPs identified by GWAS in the Labrador Retriever, a dog breed with a high disease prevalence

SNP	chr	Position	*P*	LMM	Risk Allele	f(A)	f(U)	OR	Region start-end	Genes
BICF2G630709791	1	10788643	1.64E-04	1	C	0.30	0.16	2.18	10788643-11025688	*DNAJA1, RTTN, CD226, DOK6*
BICF2S23147946	1	17290917	1.74E-04	1	G	0.28	0.15	2.19	No LD	*BCL2, PHLPP1, ZCCHC2, TNFRSF11A, KIAA1468, PPIAP1, PIGN*
BICF2P181859	1	17840093	4.32E-04	1	A	0.21	0.09	2.60	No LD	*ZCCHC2, TNFRSF11A, KIAA1468, PIGN, RNF152, CDH20*
BICF2G630712921	1	19148000	4.94E-04	1	G	0.46	0.32	1.87	18645187-19148000	*CDH20, MC4R, PMAIP1, CCBE1*
BICF2G630713147	1	19274346	4.93E-04	1	A	0.37	0.24	1.83	19253280-19274346	*MC4R, PMAIP1, CCBE1*
BICF2P818099	1	39021948	4.29E-04	3	G	0.65	0.54	1.55	No LD	*PLAGL1, SF3B5, STX11, UTRN*
BICF2S23638642	1	45229667	4.95E-04	2	G	0.18	0.07	3.07	No LD	*AKAP12, ZBTB2, C6orf211, RMND1, CCDC170, ESR1, SYNE1*
BICF2P206910	1	46405864	3.25E-04	2	C	0.14	0.04	4.05	46405864-46443183	*MYCT1, VIP, FBX05, MTRF1L, RGS17*
BICF2S22959529	1	46443183	1.58E-04	1,2	C	0.14	0.03	4.78		
BICF2P1054044	2	20002181	2.28E-04	3	G	0.85	0.77	1.74	No LD	*MTPAP, MAP3K8, BAMBI*
BICF2S23533020	2	20501149	2.47E-04	3	G	0.92	0.84	2.33	20288541-20501149	*BAMBI, MPP7*
BICF2P720951	2	62500174	4.61E-04	3	A	0.26	0.16	1.82	62252040-62500174	*GPR114, CCDC102A, DOK4,*

										COQ9, CIAPIN1, CCL17, CX3CL1, CCL22, PLLP, ARL2BP, RSPRY1, CPNE2, NLRC5, HERPUD1, SLC12A3, NUP93, MT1, MT2, MT3, MT4, BBS2, OGFOD1, AMFR, GNAO1
TIGRP2P31530	2	80046637	1.65E-04	2,3	G	0.67	0.55	1.69	80046637-80079758	*C1QB, C1QC, C1QA, EPHA8, ZBTB40, WNT4, CDC42, HSPG2, LDLRAD2, USP48, ALP, ECE1*
BICF2P247448	2	80068322	1.48E-04	2,3	G	0.68	0.55	1.67		
BICF2P1066899	2	80079758	1.65E-04	2,3	A	0.67	0.55	1.69		
BICF2G630464857	2	87862734	2.00E-04	2	G	0.14	0.04	3.69	No LD	*C1orf167, AGTRAP, DRAXIN, MAD2L2, FBXO6, FBXO44, FBXO2, PTCHD2, UBIAD1, MTOR, ANGPTL7, EXOSC10, SRM, MASP2, TARDBP, CASZ1, APITD1, DFFA, PGD, NDN, MAGEL2, MKRN3*
BICF2G630108404	3	39424250	4.96E-04	3	G	0.24	0.13	2.06	39424250-39517119	*NDN, MSGEL2, MKRN3*
BICF2S23148483	3	39469011	4.96E-04	3	G	0.24	0.13	2.06		
BICF2P866702	3	39517119	4.96E-04	3	A	0.24	0.13	2.06		
BICF2P1109077	3	48873558	4.26E-04	3	G	0.80	0.67	1.90	48873558-48880579	*MCTP2*
BICF2P1431921	3	48878860	4.26E-04	3	G	0.80	0.67	1.90		
BICF2P241884	3	48880579	4.26E-04	3	G	0.80	0.67	1.90		

BICF2P564273	3	55250188	1.07E-04	1,2,3	A	0.70	0.52	2.16	No LD	*ACAN, HAPLN3, MFGE8, ABHD2, RLBP1, FANCI, POLG, RHCG, TICRR, C3H15orf38, KIF7, PLIN1, PEX11A, WDR93, AMPN, C3H15orf38*
TIGRP2P46522	3	57698908	4.01E-04	3	C	0.70	0.54	1.91	57660110-57698908	*PDE8A, RS17, CPEB1, AP3B2, FSD2, WHAMM, HOMER2, FAM103A1, BTBD1, TM6SF1, BNC1, SH3GL3*
BICF2G630349775	3	77625147	4.26E-04	1,2	A	0.18	0.07	2.90	No LD	*TBC1D1, PGM2, RELL1, C4orf19, KIAA1239*
BICF2S2342150	3	86948527	2.91E-04	1,2	A	0.40	0.27	1.83	86948527-86974042	*STIM2, TBC1D19, CCKAR, RBPJ, SEL1L3*
BICF2S24415473	3	86974042	7.07E-05	1,2	G	0.40	0.26	1.97		
BICF2G630359517	3	91944314	3.77E-04	1,2	C	0.51	0.36	1.82	No LD	*KCNIP4, PACRGL, SLIT2*
BICF2G63058646	4	9120668	1.75E-04	1,2,3	G	0.13	0.04	3.71	No LD	*SLC35F3, KCNK1, PCNXL2*
BICF2P295392	4	14536870	4.61E-04	2	G	0.11	0.04	3.07	No LD	*BICC1, PHYHIPL, FAM13C*
BICF2G630168473	4	74924050	3.31E-04	3	G	0.30	0.18	1.96	No LD	*WDR70, NUP155, C4H5orf42, NIPBL, Q9N280, RANBP3L*
BICF2G630175389	4	84260906	5.87E-05	1,2	A	0.83	0.68	2.28	No LD	*CDH10*
BICF2G630810143	6	11130832	9.46E-05	3	A	0.44	0.32	1.72	11130832-11177149	*UPK3B, LRWD1, ALKBH4, ORAI2, PRKRIP1, SH2B2,*
BICF2G630810159	6	11177149	9.46E-05	3	A	0.44	0.32	1.72		

										CUX1, MYL10, RABL5, FIS1, ZNHIT1, PLOD3
BICF2G630810173	6	11181920	1.33E-04	3	G	0.61	0.51	1.50	11035074-11181920	*YWHAG, SRCRB4D, ZP3, UPK3B, LRWD1, ALKBH4, ORAI2, PRKRIP1, SH2B2, CUX1, MYL10, RABL5, FIS1, ZNHIT1, PLOD3, MOGAT3*
BICF2P170661	6	11439931	3.39E-04	3	A	0.31	0.22	1.57	No LD	*CUX1, MYL10, RABL5, FIS1, ZNHIT1, PLOD3, MOGAT3, AP1S1, SERPINE1, MUC3A, ACHE, SLC12A9, ACHE, SRRT, EPHB4*
BICF2P1354767	6	11462695	3.02E-04	3	G	0.48	0.38	1.53	11106977-11462695	*UPK3B, LRWD1, ORAI2, PRKRIP1, SH2B2, CUX1, MYL10, RABL5, FIS1, ZNHIT1, PLOD3, MOGAT3, Q7YSA1, MUC3A, ACHE, SRRT, SLC12A9, EPHB4*
BICF2P205255	6	11484772	4.75E-04	3	G	0.54	0.45	1.43	No LD	*CUX1, MYL10, RABL5, FIS1, ZNHIT1, PLOD3, MOGAT3, AP1S1, SERPINE1, MUC3A, ACHE, SLC12A9, ACHE, SRRT, EPHB4*

BICF2P1358119	6	13131171	3.74E-04	3	C	0.44	0.34	1.54	13131171-13182379	*AZGP1, GJC3, TRIM4, OR2AE1, CYP3A12, ZNF498, ZNF655, FAM200A, ZKSCAN5, ZNF394, ZNF789, ATP5J2, CPSF4, PDAP1, ARPC1B, KPNA7, SMURF1, TRRAP, TMEM130*
BICF2S23324965	6	14077648	3.36E-05	3	G	0.68	0.60	1.42	14077648-14092057	*TRRAP, TMEM130, NPTX2, BAIAP2L1, BRI3, TECPR1, LMTK2, PMS2, EIF2AK1, ANKRD61, USP42, CYTH3*
BICF2S22961650	6	14092057	1.53E-04	3	G	0.68	0.61	1.35		
BICF2P498515	6	75848537	7.89E-05	1,2,3	A	0.16	0.06	3.11	No LD	*LRRIQ3*
BICF2P1072682	7	53407178	4.09E-04	1,2,3	C	0.28	0.14	2.42	No LD	*None*
BICF2P1090079	7	64389761	1.56E-04	1,3	C	0.47	0.31	1.94	No LD	*CDH2, CHST9*
BICF2P1208798	9	12671217	5.49E-05	1,2	G	0.56	0.36	2.27	No LD	*EFCAB13, ITGB3, MYL4, CDC27, KANSL1, MAPT, SPPL2C, CRHR1, NSF, WNT3*
BICF2P890246	9	53427907	3.23E-05	1,2	A	0.16	0.36	2.99	53427907-53432248	*SOHLH1, ALL2, LCN9, GLT6D1, LCN1, SURF1, SURF2, SURF4, MED22, C9H9orf96, REXO4, ADAMTS13, CACFD1, SLC2A6, ADAMTSL2, DOPO, SARDH, VAV2, WDR5, RXRA*
BICF2P139678	9	53432248	1.75E-04	1,2	A	0.83	0.65	2.71		

BICF2S23113199	10	46246942	3.57E-04	1,3	A	0.63	0.46	1.95	No LD	*AFF3, REV1, EIF5B, TXNDC9, LYG1, LYG2, MRPL30, MITD1, LIPT1, TSGA10*
BICF2P401973	10	65344772	3.79E-04	1	G	0.84	0.71	2.23	No LD	*FAM161A, CCT4, COMMD1, B3GNT2, TMEM17, EHBP1*
BICF2P454456	11	32175491	4.77E-04	3	A	0.19	0.13	1.62	31831896-32175491	*C11H9orf123, PTPRD*
BICF2P50610	11	32270617	2.75E-05	3	A	0.29	0.19	1.70	31939564-32270617	*C11H9orf123, PTPRD*
BICF2P531097	11	32908558	3.58E-04	3	A	0.44	0.29	1.92	32908558-32922914	*PTPRD*
BICF2P1290820	11	32922914	4.19E-04	3	G	0.44	0.30	1.91		
BICF2P65003	12	40691540	3.15E-04	2	G	0.71	0.61	1.61	40691540-41066621	*SENP6, MYO6, IMPG1*
BICF2G630606359	13	13352804	4.61E-04	2	G	0.69	0.57	1.67	13352804-13503950	*NUDCD1, PKHD1L1, EBAG9, SYBU, KCNV1*
BICF2S23620879	14	10265645	4.05E-04	3	C	0.5	0.37	1.71	9796003-10265645	*COPG2, MEST, CEP41, CPA1, CPA4, SSMEM1, TMEM209, KLHDC10, UBE2H, NRF1, FAM40B, AHCYL2, SMO, TSPAN33, TNPO3, IRF5, KCP, ATP6V1F, FLNC*
BICF2G630519882	14	11686985	4.99E-04	3	A	0.49	0.36	1.74	11675474-11686985	*SND1, LRRC4, PAX4, ARF5, GCC1, ZNF800, GRM8*
BICF2P594418	15	58424953	4.48E-04	2	A	0.19	0.10	2.07	No LD	*FAM198B, TMEM144, RXFP1,*

										ETFDH, PPID, C4orf46, FNIP2
BICF2G630422966	15	58852255	4.41E-04	3	A	0.41	0.28	1.80	58852255-58978372	*FAM198B, TMEM144, RXFP1, ETFDH, PPID, FNIP2, C4orf46, C4orf45, RAPGEF2*
BICF2G630422956	15	58891376	4.41E-04	3	A	0.41	0.28	1.80		
BICF2G630422900	15	58967776	2.04E-04	3	G	0.37	0.24	1.87		
BICF2G630422895	15	58978372	1.69E-04	3	A	0.37	0.24	1.89		
BICF2P880005	17	20749191	2.22E-04	1,2	G	0.44	0.31	1.75	No LD	*KLHL29, ATAD2B*
BICF2P1121006	18	54279578	1.11E-04	1,2,3	A	0.63	0.42	2.28	No LD	*CCS, CCDC87, CTSF, ZDHHC24, PELI3, MRPL11, SLC29A2, B3GNT1, BRMS1, RIN1, CD248, TMEM151A, YIF1A, CNIH2, KLC2, PACS1, SF3B2, GAL3ST3, CATSPER1, BANF1, EIF1AD, SART1, EFEMP2, MUS81, FIBP, FOSL1, CCDC85B, CTSW, SNX32, OVOL1, AP5B1, RNASEH2C, KAT5, A1XFH4, SIPA1, PCNXL3, MAP3K11, EHBP1L1, LTBP3, SCYL1*
BICF2P888055	20	13815084	3.25E-04	3	A	0.73	0.62	1.71	No LD	*GRM7*
BICF2P582174	20	14124824	3.76E-04	3	C	0.82	0.73	1.61	14118014-14124824	*GRM7*
TIGRP2P270462	20	15036973	8.51E-05	3	G	0.85	0.75	1.88	14838270-15053718	*EDEM1, ARL8B*
BICF2P716829	20	15048191	9.85E-05	3	G	0.85	0.75	1.86		

BICF2P1462185	20	15053718	4.90E-05	3	A	0.85	0.74	1.90		
BICF2P178583	20	30190042	1.41E-04	1,2	G	0.48	0.31	2.09	30039696-30190042	*ADAMTS9, PRICKLE2, PSMD6, ATXN7, THOC7, SNTN, C3orf49, SYNPR*
BICF2S2328420	20	51150968	2.77E-04	3	G	0.75	0.60	1.96	No LD	*ADGRE3, ADGRE2, ZNF333, CLEC17A, NDUFB7, TECR, DNAJB1, GIPC1, PTGER1, PKN1, DDX39A, ADGRE5, PRKACA, C19orf67, PALM3, IL27RA, RFX1, DCAF15, PODNL1, CC2D1A, C20H19orf57, NANOS3, ZSWIM4, MRI1*
BICF2P420488	20	52326317	3.84E-04	3	G	0.85	0.75	1.84	51873051-52326317	*C19orf57, PRKACA, PALM3, IL27RA, RFX1, DCAF15, PODNL1, CC2D1A, C20H19orf57, NANOS3, ZSWIM4, MRI1, CCDC130, CACNA1A, NACC1, IER2, STX10, TRMT1, LYL1, NFIX, DAND5, RAD23A, CALR, SYCE2, RTBDN, KLF1, DNASE2, GCDH, RNASEH2A, PRDX2, JUNB, HOOK2, BEST2,*

										TNPO2, FBXW9, DHPS, WDR83, WDR83OS, MAN2B1, ZNF791, ACP5, ELOF1, ACP5
TIGRP2P277002	20	55563965	2.25E-04	1,2	A	0.25	0.10	2.98	No LD	*INSR, ARHGEF18, PEX11G, ZNF358, MCOLN1, CAMSAP3, XAB2, STXBP2, RETN, TRAPPC5, FCER2, CLEC4G, CD209, EVI5L, LRRC8E, MAP2K7, SNAPC2, CCL25, TIMM44, ELAVL1, FBN3, CERS4, CD320, KANK3, ANGPTL4, RAB11B, MARCH2, HNRNPM, PRAM1, ZNF414, MYO1F*
BICF2P111342	21	7150110	1.25E-04	1,2	A	0.31	0.18	2.04	No LD	*None*
BICF2G630658881	21	7582214	1.09E-04	1,2,3	G	0.49	0.32	2.12	7582214-8382709	*JRKL, CCDC82, MAML2, MTMR2, CEP57, FAM76B, SESN3*
BICF2G630658668	21	8205285	4.03E-04	3	G	0.49	0.32	2.00		
BICF2G630658620	21	8382709	2.90E-04	3	G	0.50	0.33	2.02		
BICF2G630658768	21	8033283	4.09E-04	1,2	C	0.27	0.14	2.28	8033283-8061623	*JRKL, CCDC82, MAML2, MTMR2, CEP57, FAM76B*
BICF2G630658756	21	8040746	4.73E-04	1	A	0.26	0.13	2.30		
BICF2G630658723	21	8061623	4.09E-04	1,2	G	0.27	0.14	2.28		
BICF2S2442023	21	43533585	3.19E-04	3	G	0.50	0.34	1.93	43507320-43533585	*NUCB2, NCR3LG1, KCNJ11, ABCC8, USH1C, OTOG, MYOD1, KCNC1,*

										U6, TPH1, SERGEF, SAAL1, SAA1, HPS5, GTF2H1
BICF2S2361376	21	43752575	1.76E-04	1,2,3	A	0.60	0.42	1.97	43752575-43808389	*USH1C, OTOG, MYOD1, KCNC1, U6, B6EY10, SAAL1, SAA1, HPS5, GTFSH1, LDHC, TSG101, UEVLD, SPTY2D1, TMEM86A, PTPN5*
BICF2P321064	21	44627903	1.66E-04	1,2,3	G	0.38	0.24	1.99	No LD	*TSG101, UEVLD, SPTY2D1, TMEM86A, PTPN5, ZDHHC13, CSRP3, E2F8, NAV2*
TIGRP2P293361	22	42354230	2.27E-04	2	A	0.49	0.36	1.74	No LD	*SLITRK5*
TIGRP2P297337	22	58201452	1.08E-04	1,2,3	A	0.44	0.27	2.20	No LD	*EFNB2, ARGLU1*
BICF2G630375268	23	33376383	3.48E-04	3	A	0.74	0.63	1.75	No LD	*TMEM108, BFSP2, CDV3, TOPBP1, TF, RAB6B, SLCO2A1*
BICF2S23730962	23	53809871	2.93E-04	2	A	0.87	0.78	1.88	No LD	*TIPARP, LEKR1, CCNL1, VEPH1, PTX3*
BICF2G630502225	24	23992936	4.86E-04	2	G	0.92	0.81	2.84	No LD	*ZCCHC3, C20orf96, DEFB132, DEFB128, B0FF14, DEFB126, DEFB125, DEFB118, DEFB116, DEFB121, DB119, DEFB122, BOFF10, BCL-XL,*

										HM13CO4I2, TPX2, MYLK2, DUSP15, TTLL9, PDRG1, XKR7, HCK, TM9SF, C20orf160
BICF2G630500835	24	29648925	1.29E-04	2,3	A	0.79	0.67	1.90	No LD	*CTNNBL1, VSTM2L, TTI1, RPRD1B, TGM2, KIAA1755, BPI, LBP, RALGAPB, ADIG, SLC32A1, ACTR5*
BICF2P544126	24	29772193	4.09E-05	3	G	0.94	0.87	2.28	29772193-29794411	*CTNNBL1, VSTM2L, TTI1, RPRD1B, TGM2, KIAA1755, BPI, LBP, RALGAPB, ADIG, SLC32A1, ACTR5, PPP1R16B, FAM83D*
BICF2S24111418	24	29794411	4.09E-05	3	A	0.94	0.87	2.28		
BICF2G630500368	24	30241088	2.76E-07	1,2,3	G	0.83	0.66	2.56	30241088-30245795	*BPI, LBP, RALGAPB, SLC32A1, ADIG, ACTR5, PPP1R16B, FAM83D, DHX35*
BICF2G630500363	24	30245795	1.82E-06	1,2,3	G	0.80	0.62	2.44		
BICF2G630799191	26	22848912	1.33E-04	3	G	0.61	0.45	1.92	No LD	*ADRBK2, MYO18B, SEZ6L, ASPHD2, HPS4, SRRD, TFP11, TPST2, CRYBB1, CRBA4*
BICF2P792911	26	22894961	8.55E-05	1,2,3	G	0.44	0.27	2.14	No LD	*ADRBK2, MYO18B, SEZ6L, ASPHD2, HPS4, SRRD, TFP11, TPST2, CRYBB1, CRBA4*
BICF2S2356299	27	30557856	2.21E-05	2,3	A	0.43	0.27	2.03	No LD	*AEBP2, PLEKHA5*

BICF2P1332722	27	30603252	2.19E-04	1,2	G	0.78	0.60	2.33	No LD	*AEBP2, PLEKHA5*
BICF2P1047447	27	31108106	2.80E-04	3	A	0.52	0.38	1.75	No LD	*AEBP2, PLEKHA5, CAPZA3, PLCZ1, PIK3C2G*
BICF2P487060	27	33778510	4.55E-04	3	C	0.40	0.27	1.81	33778510-33809600	*MGST1, SLC15A5, DERA, STRAP, EPS8, PTPRO*
BICF2P599881	27	35600038	2.66E-04	3	G	0.85	0.74	1.97	No LD	*PLBD1, ATF7IP, NMDE2*
BICF2P1410038	27	37697040	3.33E-04	3	C	0.70	0.56	1.82	No LD	*BCL2L14, ETV6, TAS2R42, CAFA-T2R67, CAFA-T2R43, CAFA-T2R12, TAS2R10, TAS2R7, CSDA, STYK1, MAGOHB, LY49*
BICF2S23535135	27	37814333	1.13E-04	3	A	0.31	0.20	1.83	No LD	*ETV6, TAS2R42, CAFA-T2R67, CAFA-T2R43, CAFA-T2R12, TAS2R10, TAS2R7, CSDA, STYK1, MAGOHB, LY49*
TIGRP2P355298	27	39134291	1.31E-04	3	G	0.74	0.57	2.16	No LD	*KLRK1, KLRD1, GABARAPL1, TMEM52B, OLR1, CLEC7A, CLEC1B, CLEC9A, CLEC12B, CLEC12A, CLEC2B, KLRF1, CD69, CLEC2D, KLRB1*
BICF2S23255928	27	39211186	1.10E-04	2,3	A	0.23	0.13	2.07	39211186-39217437	*KLRD1, GABARAPL1,*
BICF2P526639	27	39217437	4.12E-05	2,3	G	0.23	0.12	2.18		

										TMEM52B, OLR1, CLEC7A, CLEC1B, CLEC12B, CLEC12A, CLEC2B, KLRF1, CD69, CLEC2D, KLRB1
BICF2S23152419	27	39428263	1.77E-05	3	A	0.79	0.64	2.13	39428263-39445306	*CLEC1B, CLEC12A, CLEC2B, KLRF1, CD69, CLEC2D, KLRB1, A2M, KLRG1, M6PR, PHC1, RIMKLB*
BICF2P491441	27	39434491	3.34E-04	3	G	0.78	0.63	2.06		
TIGRP2P355396	27	39445306	3.34E-04	3	A	0.78	0.63	2.06		
BICF2P337576	27	39463031	2.35E-04	3	G	0.70	0.55	1.92	39346073-39511019	*OLR1, CLEC7A, CLEC1B, CLEC12B, CLEC12A, CLEC2B, KLRF1, CD69, CLEC2D, KLRB1, A2M, KLRG1, M6PR, PHC1, RIMKLB*
BICF2P794117	27	39511019	3.76E-04	3	C	0.71	0.56	2.00		
BICF2P155064	27	39526004	4.68E-04	3	G	0.21	0.14	4.78	No LD	*CLEC12A, CLEC2B, KLRF1, CD69, CLEC2D, KLRB1, A2M, KLRG1, M6PR, PHC1, RIMKLB*
BICF2P992747	27	39580957	1.65E-04	3	A	0.77	0.62	2.07	No LD	*CLEC2B, KLRF1, CD69, CLEC2D, KLRB1, A2M, KLRG1, M6PR, PHC1, RIMKLB, MFAP5*
BICF2S23652189	27	39644847	3.99E-04	3	G	0.31	0.21	1.64	39606871-39644847	*AICDA, APOBEC1,*

										DPPA3
TIGRP2P362234	28	41376035	3.71E-04	2	G	0.87	0.74	2.37	41376035-41377128	*MGMT, EBF3, GLRX3*
BICF2S23346408	28	41377128	1.25E-04	1,2	A	0.87	0.73	2.53		
BICF2S23713161	29	20562935	4.59E-04	2	G	0.75	0.63	1.74	No LD	*PREX2, C29H8orf34*
BICF2S23410873	29	20672864	2.18E-04	2,3	C	0.82	0.66	2.35	20672864-20703202	*PREX2, C29H8orf34*
BICF2P1135545	29	20703202	2.81E-04	2,3	A	0.82	0.67	2.31		
BICF2P483191	29	21601273	2.31E-05	1,2,3	C	0.73	0.51	2.54	No LD	*C29H8orf34, SULF1, SLCO5A1*
BICF2P139173	29	22067666	3.59E-04	2	A	0.50	0.36	1.79	22050835-22191229	*SULF1, SLCO5A1, VTI1B, PROM14, NCOA2, TRAM1*
BICF2P361907	29	22191229	3.47E-04	2	G	0.50	0.36	1.75		
BICF2P456086	29	23130206	4.85E-04	3	A	0.88	0.71	3.04	No LD	*TRAM1, LACTB2, XKR9, EYA1 TRAM1, LACTB2, XKR9, EYA1*
BICF2P662502	29	26040013	3.24E-04	1,2	G	0.90	0.73	2.87	No LD	*JPH1, GDAP1, PI15, CRISPLD1*
BICF2G630412697	30	3126573	7.22E-05	1,3	G	0.96	0.86	4.23	No LD	*OR4K2, OR4K1, ORFN5*
BICF2S2356993	31	12920807	2.25E-04	2,3	A	0.25	0.14	2.04	No LD	*ROBO2*
BICF2P287265	31	30555902	4.38E-04	3	G	0.96	0.87	3.38	No LD	*MIS18A, MRAP, URB1, FAM176C, C31H21orf59, SYNJ1, GCFC1, OLIG2, OLIG1, SC5A3, SUB1, IL10RB*
BICF2S23054250	35	26868731	2.20E-04	2,3	A	0.49	0.33	1.97	No LD	*LRRC16A, SCGN, HIST1H2AA, HIST1H2BA, SLC17A4, SLC17A1, SLC17A2, TRIM38, HFE, HIST1H4E, HIST1H1C,*

	37									*HIST1H1A, HIST1H3C, HIST1H3A, HIST1H2AE, HIST1H2BB, HIST1H1T, HIST1H2BC, HIST1H2AC, HIST4H4, HIST2H2AB, HIST1H4F, HIST1H2BG, HIST1H4L, BTN1A1, HMGN4*
BICF2P1086740	37	26916351	4.34E-04	2	G	0.41	0.24	2.18	No LD	*SMARCAL1, IGFBP2, IGFBP5, TNP1*
BICF2P708698	37	26924473	1.53E-04	2	A	0.63	0.45	2.06	No LD	*SMARCAL1, IGFBP2, IGFBP5, TNP1*

Note: OR odds ratio calculated from PLINK [32]. LMM Linear mixed model 1 – GCTA [33], 2 – GEMMA [34], 3 – PUMA [35]. f(A) and f(U) represent the frequency of the risk allele in case and control dogs, respectively. SNP position and genomic regions are based on CanFam 2.0. The gene list was created using UCSC Genome Browser after LiftOver to CanFam 3.1 and the addition of 500kb flanking regions.
