# Supplementary material for: Genome-wide association analysis in dogs implicates 99 loci as risk variants for anterior cruciate ligament rupture
Source: PLoS One. 2017 Apr 5;12(4):e0173810. doi: 10.1371/journal.pone.0173810 (PMC5381864; doi:10.1371/journal.pone.0173810)
Supplement: S2 Table — (PDF) [file pone.0173810.s003.pdf]

Table S2 | Statistical Power and odds ratio correction for anterior cruciate ligament rupture GWAS risk loci identified by GEMMA in the Labrador Retriever

SNP	Chr	MAF	Beta	Significance	OR	f(u)	Power	Corrected OR	Corrected Power	INPower Estimated number of loci
BICF2S23638642	1	0.12	0.23	4.95E-04	3.07	0.07	0.99	1.38	0.19	6.1
BICF2S22959529	1	0.08	0.31	1.58E-04	4.78	0.03	0.99	1.83	0.30	3.9
BICF2P247448	2	0.40	-0.18	1.48E-04	1.67	0.55	0.84	1.23	0.22	2.6
BICF2G630464857	2	0.08	0.30	2.00E-04	3.69	0.04	0.98	1.58	0.23	3.7
BICF2P564273	3	0.41	-0.17	1.07E-04	2.16	0.52	0.99	1.47	0.61	3.5
BICF2G630349775	3	0.12	0.24	4.26E-04	2.90	0.07	0.98	1.37	0.18	5.5
BICF2S24415473	3	0.32	0.19	7.07E-05	1.97	0.26	0.96	1.50	0.59	2.0
BICF2G630359517	3	0.42	0.15	3.77E-04	1.82	0.36	0.94	1.20	0.18	6.2
BICF2G63058646	4	0.08	0.30	1.76E-04	3.71	0.04	0.98	1.62	0.25	4.1
BICF2P295392	4	0.07	0.29	4.61E-04	3.07	0.04	0.91	1.39	0.14	5.9
BICF2G630175389	4	0.26	-0.20	5.87E-05	2.28	0.68	0.98	1.69	0.77	2.3
BICF2P498515	6	0.10	0.29	7.89E-05	3.11	0.06	0.98	1.93	0.57	3.4
BICF2P1072682	7	0.20	0.20	4.09E-04	2.42	0.14	0.99	1.30	0.21	4.1
BICF2P1208798	9	0.44	0.21	5.49E-05	2.27	0.36	1.00	1.70	0.87	1.9
BICF2P890246	9	0.28	-0.21	3.23E-05	2.99	0.36	1.00	2.19	1.00	2.5
BICF2P65003	12	0.35	-0.20	3.15E-04	1.61	0.61	0.75	1.16	0.13	2.5
BICF2G630606359	13	0.38	-0.16	4.61E-04	1.67	0.57	0.83	1.16	0.14	4.8
BICF2P594418	15	0.14	0.22	4.48E-04	2.07	0.10	0.84	1.24	0.13	4.6
BICF2P880005	17	0.37	0.17	2.22E-04	1.75	0.31	0.89	1.21	0.19	4.0
BICF2P1121006	18	0.49	-0.21	1.11E-04	2.28	0.42	1.00	1.50	0.66	1.6
BICF2P178583	20	0.38	0.18	1.41E-04	2.09	0.31	0.99	1.36	0.41	3.4
TIGRP2P277002	20	0.16	0.23	2.25E-04	2.98	0.10	1.00	1.45	0.30	3.0
BICF2P111342	21	0.06	0.20	1.25E-04	2.04	0.18	0.95	1.38	0.34	3.1
BICF2G630658881	21	0.39	0.19	1.09E-04	2.12	0.32	0.99	1.45	0.56	2.0
BICF2G630658768	21	0.19	0.21	4.09E-04	2.28	0.14	0.97	1.28	0.19	3.9
BICF2S2361376	21	0.49	0.17	1.76E-04	1.97	0.42	0.98	1.28	0.30	3.8
BICF2P321064	21	0.30	0.18	1.66E-04	1.99	0.24	0.96	1.29	0.27	3.7

TIGRP2P293361	22	0.42	0.17	2.27E-04	1.74	0.36	0.90	1.21	0.20	4.0
TIGRP2P297337	22	0.34	0.19	1.08E-04	2.20	0.27	0.99	1.48	0.57	2.3
BICF2S23730962	23	0.19	-0.22	2.93E-04	1.88	0.78	0.79	1.22	0.15	3.8
BICF2G630502225	24	0.14	-0.22	4.86E-04	2.84	0.81	0.98	1.35	0.25	4.4
BICF2G630500835	24	0.28	-0.17	1.29E-04	1.90	0.67	0.91	1.33	0.33	4.4
BICF2G630500368	24	0.27	-0.26	2.76E-07	2.56	0.66	1.00	2.44	1.00	0.8
BICF2P792911	26	0.34	0.19	8.55E-05	2.14	0.27	0.99	1.53	0.65	2.2
BICF2S2356299	27	0.34	0.17	2.21E-05	2.03	0.27	0.98	1.71	0.84	4.2
BICF2P1332722	27	0.32	-0.18	2.19E-04	2.33	0.60	1.00	1.34	0.38	3.8
BICF2P526639	27	0.17	0.23	4.12E-05	2.18	0.12	0.93	1.71	0.63	3.5
BICF2S23346408	28	0.21	-0.22	1.25E-04	2.53	0.73	0.99	1.53	0.55	2.2
BICF2S23713161	29	0.32	-0.16	4.59E-04	1.74	0.63	0.85	1.18	0.15	6.2
BICF2S23410873	29	0.27	-0.18	2.18E-04	2.35	0.66	0.99	1.34	0.35	4.6
BICF2P483191	29	0.39	-0.20	2.31E-05	2.54	0.51	1.00	2.02	0.98	2.3
BICF2P361907	29	0.42	0.18	3.47E-04	1.75	0.36	0.90	1.19	0.17	2.8
BICF2P662502	29	0.20	-0.19	3.24E-04	2.87	0.73	1.00	1.39	0.37	4.9
BICF2S2356993	31	0.19	0.20	2.25E-04	2.04	0.14	0.91	1.27	0.18	4.7
BICF2S23054250	35	0.39	0.16	2.20E-04	1.97	0.33	0.98	1.26	0.26	4.1
BICF2P1086740	37	0.31	0.17	4.34E-04	2.18	0.24	0.99	1.26	0.23	4.1
BICF2P708698	37	0.48	-0.16	1.53E-04	2.06	0.45	0.99	1.33	0.39	4.1

Note: MAF minor allele frequency, OR odds ratio, and f(u) frequency of risk allele in control dogs calculated from PLINK [32]. Corrected OR was calculated using an approximate conditional likelihood approach [41]. GWAS data were derived using GEMMA [34]. For each risk locus detected by GEMMA, the total number of risk loci was estimated using INPower [40].
