# Supplementary material for: Genome-wide association analysis in dogs implicates 99 loci as risk variants for anterior cruciate ligament rupture
Source: PLoS One. 2017 Apr 5;12(4):e0173810. doi: 10.1371/journal.pone.0173810 (PMC5381864; doi:10.1371/journal.pone.0173810)
Supplement: S3 Table — (PDF) [file pone.0173810.s004.pdf]

Table S3 | Estimation of the expected number of loci to be discovered in a future GWAS of ACL rupture in the Labrador Retriever using INPower

Sample size	Expected number of loci to be discovered	Probability of detecting at least the expected number of loci
250	0.0	0.00 (0 loci)
500	0.0	0.02 (1 locus)
750	0.1	0.06 (1 locus)
1,000	0.2	0.15 (1 locus)
1,250	0.3	0.29 (1 locus)
1,500	0.6	0.47 (1 locus)
1,750	1.1	0.66 (1 locus)
2,000	1.7	0.51 (2 loci)
2,250	2.6	0.25 (3 loci)
2,500	3.7	0.52 (4 loci)

Note: Power calculations were performed with INPower [40].
